# Quantitative analysis of the formation of nucleoprotein complexes between HIV-1 Gag protein and genomic RNA using transmission electron microscopy

**DOI:** 10.1016/j.jbc.2021.101500

**Published:** 2021-12-17

**Authors:** Stéphanie Durand, Florian Seigneuret, Julien Burlaud-Gaillard, Roxane Lemoine, Marc-Florent Tassi, Alain Moreau, Marylène Mougel, Philippe Roingeard, Clovis Tauber, Hugues de Rocquigny

**Affiliations:** 1 Morphogenesis and Antigenicity of HIV and Hepatitis Viruses, Inserm – U1259 MAVIVH, Bretonneau Hospital, Tours Cedex 1, France; 2 Microscopy IBiSA Platform, PPF ASB, University of Tours and CHRU of Tours, Tours Cedex 1, France; 3 B Cell Ressources Platform, EA4245 “Transplantation, Immunology and Inflammation”, University of Tours, Tours Cedex 1, France; 4 Équipe R2D2 Retroviral RNA Dynamics and Delivery, IRIM, CNRS UMR9004, University of Montpellier, Montpellier, France; 5 UMR U1253 iBrain, Inserm, University of Tours, Tours Cedex 1, France

**Keywords:** HIV-1, assembly, Gag, genomic RNA, transmission electron microscopy, morphometric TEM analysis, BSA, bovine serum albumin, eGFP, enhanced GFP, gRNA, genomic RNA, PM, plasma membrane, PT, posttransfection, RSV, Rous Sarcoma Virus, SL, stem loop, TEM, Transmission Electron Microscopy, ZF, zinc finger

## Abstract

In HIV, the polyprotein precursor Gag orchestrates the formation of the viral capsid. In the current view of this viral assembly, Gag forms low-order oligomers that bind to the viral genomic RNA triggering the formation of high-ordered ribonucleoprotein complexes. However, this assembly model was established using biochemical or imaging methods that do not describe the cellular location hosting Gag–gRNA complex nor distinguish gRNA packaging in single particles. Here, we studied the intracellular localization of these complexes by electron microscopy and monitored the distances between the two partners by morphometric analysis of gold beads specifically labeling Gag and gRNA. We found that formation of these viral clusters occurred shortly after the nuclear export of the gRNA. During their transport to the plasma membrane, the distance between Gag and gRNA decreases together with an increase of gRNA packaging. Point mutations in the zinc finger patterns of the nucleocapsid domain of Gag caused an increase in the distance between Gag and gRNA as well as a sharp decrease of gRNA packaged into virions. Finally, we show that removal of stem loop 1 of the 5′-untranslated region does not interfere with gRNA packaging, whereas combined with the removal of stem loop 3 is sufficient to decrease but not abolish Gag-gRNA cluster formation and gRNA packaging. In conclusion, this morphometric analysis of Gag-gRNA cluster formation sheds new light on HIV-1 assembly that can be used to describe at nanoscale resolution other viral assembly steps involving RNA or protein–protein interactions.

Production of HIV-1 particles by infected cells is a complex process coordinated by Gag and GagPol polyprotein precursors that promote the specific encapsidation of the psi containing genomic RNA (gRNA). In HIV, unspliced gRNA is exported from the nucleus through a REV-dependent active transport. After translation, Gag assembly begins with binding to gRNA resulting in gRNA dimerization and the formation of Gag oligomers. During virus assembly, Gag is processed by the viral protease generating the MAp17, CAp24, NCp7, and p6 structural proteins found in infectious mature viral particles soon after budding (1). The current model for HIV-1 assembly stipulates that Gag oligomerization results from CA–CA interactions along with Gag binding to the gRNA mediated by the NC domain (referred to as GagNC), causing the formation of Gag–gRNA ribonucleoprotein complexes (2). This oligomerization process also induces a structural modification of MA, exposing its N-terminal myristate that, together with the MA highly basic region, target the Gag–gRNA oligomers to the plasma membrane (PM) enriched in PI(4,5)P2 lipids (3). This model is supported by biochemical findings showing that the binding of low-order Gag oligomers to the gRNA occurs in the cytoplasm and then traffic to the plasma membrane where thousands of Gag molecules are added to complete assembly resulting in the formation of immature virions (4). Cytoplasmic localization of Gag–gRNA nucleoprotein complexes and their traffic to the PM were also confirmed by the colocalization of the two partners as observed by optical microscopy (5, 6, 7, 8, 9, 10) and by monitoring their interaction using quantitative fluorescent microscopies (11, 12). Gag-gRNA trafficking was proposed to rely either on the free diffusion of the complexes or through an endosomal pathway depending on the cells used (5, 8, 9, 13, 14, 15, 16). It should be noted here that in another retrovirus such as Rous Sarcoma Virus (RSV), it was proposed that in the absence of transacting protein like REV, the nuclear cycling property of Gag could facilitate the nuclear export and the encapsidation of gRNA in virions (17).

In HIV assembly model, the role of GagNC is central because it directs the specific recruitment of the gRNA with its concomitant dimerization and contributes to the core condensation during or soon after virus budding (18). Except for Spumaviruses, all retroviral NC’s harbor one or two conserved CCHC domains referred to as zinc fingers (ZF) that bind zinc ion with high affinity (19). NMR studies on HIV-1 NC revealed that each domain folds into a zinc knuckle (20, 21) and that the two are close to each other forming a hydrophobic platform essential for RNA binding (22). Mutating the conserved C or H residue for S or A caused a drastic decrease in the affinity for Zn^2+^ impacting on NCp7 folding and gRNA packaging (23, 24). NC binds with high affinity to sequences located in the Psi sequence of the 5′UTR of the gRNA (25, 26, 27, 28, 29). Nevertheless, similar affinities were observed under physiological conditions for cellular RNAs (30, 31), suggesting that other parameters are involved for the selective packaging of gRNA among the bulk of cellular RNAs. One possibility could be a high efficiency of Gag to nucleate virus-like particle(s) formation promoted by viral Psi-containing gRNA (32, 33). Else, the kinetic of Gag to reach the PM would be impacted by zinc finger deletions that cause a strong delay in Gag and Gag-gRNA trafficking to the PM (11, 34), leading to intracellular Gag accumulation and particle budding defects (15, 35, 36, 37). However, all of this work on HIV assembly was carried out using biochemical or imaging methods that do not distinguish gRNA packaging in single particles nor describe the cellular location harboring this interaction. To better characterize the cellular environment of HIV morphogenesis and the role of GagNC in the formation of Gag–gRNA complexes, we used the higher resolution of transmission electronic microscopy (TEM). A morphometric analysis of the gold bead labeling Gag and gRNA on TEM micrographs allowed quantification of the distance between Gag and gRNA. Interestingly, this distance decreased as a function of time together with an increasing accumulation of ribonucleoprotein complexes toward the PM suggesting a condensation of the vRNP(ribonucleoprotein). The shortest distances between Gag and gRNA were obtained with Gag-WT, Gag-G2A, and Gag-Δp6, whereas the largest distances were found when GagNC was deleted or mutated or when both SL1 and SL3 were deleted. As a matter of fact, almost 25% of virions contained gRNA at 24 h posttransfection (PT) and this was drastically reduced to a few % when the ZFs were deleted or mutated. All together, these results show that cytoplasmic Gag–gRNA complexes traffic toward the PM as a function of time and the quantitative analysis of our TEM images reveal that this spatio-temporal distribution is concomitant with a condensation of the nucleoprotein complex that in turn is essential for a specific gRNA packaging.

## Results

### Gag and the gRNA form clusters in the cytoplasm that translocate to the PM as a function of time

**Figure 1:**
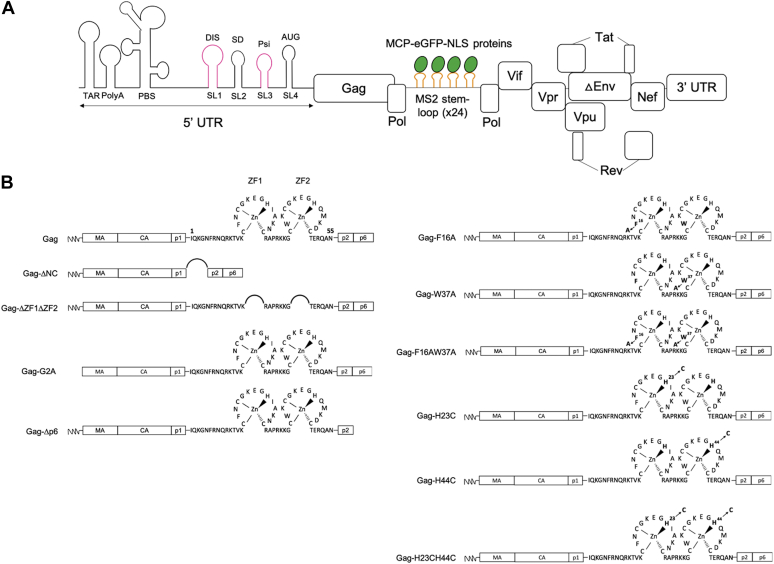
**Scheme of HIV-1 gRNA-MS2-Δenv and Gag or Gag derivatives.***A*, HIV-1 gRNA-MS2-Δenv is expressed from pNL4.3-MS2-Δenv plasmid and is translated under the control of HIV-1 LTR promotor in all viral genes except *pol* and *env*. The 5′ UTR contains highly structured regions important for HIV-1 replication, notably four stem-loops (SL1–SL4). This gRNA-MS2-Δenv is detected by the insertion of 24 stem-loops (*orange symbols*) of the MS2 bacteriophage in the pol gene interacting with MCP-eGFP-NLS proteins (*green circles*). *B*, HIV-1 Gag domains are shown as empty boxes, whereas NC is represented by its primary sequence. Deletion of each domain is symbolized by a bridge that links the flanking sequences. The substitution mutants are shown with their position and the replacing amino acid. eGFP, enhanced GFP; gRNA, genomic RNA.

To monitor the colocalization of HIV-1 Gag and gRNA at high resolution, we used TEM on HeLa cells transfected by pNL4.3-MS2-Δenv and pMCP-eGFP-NLS (Fig. 1*A*). Gag was detected by anti-p24 antibody and the gRNA by the use of MCP-eGFP-NLS, as already described (Fig. S1*A*) (38, 39). Note that the pNL4.3-MS2-Δenv plasmid expressed the recombinant gRNA-MS2-Δenv that contains *cis* elements essential for gRNA encapsidation and encodes for Gag to avoid any perturbation of Gag–gRNA complex using Gag in *trans* (13).

**Figure 2:**
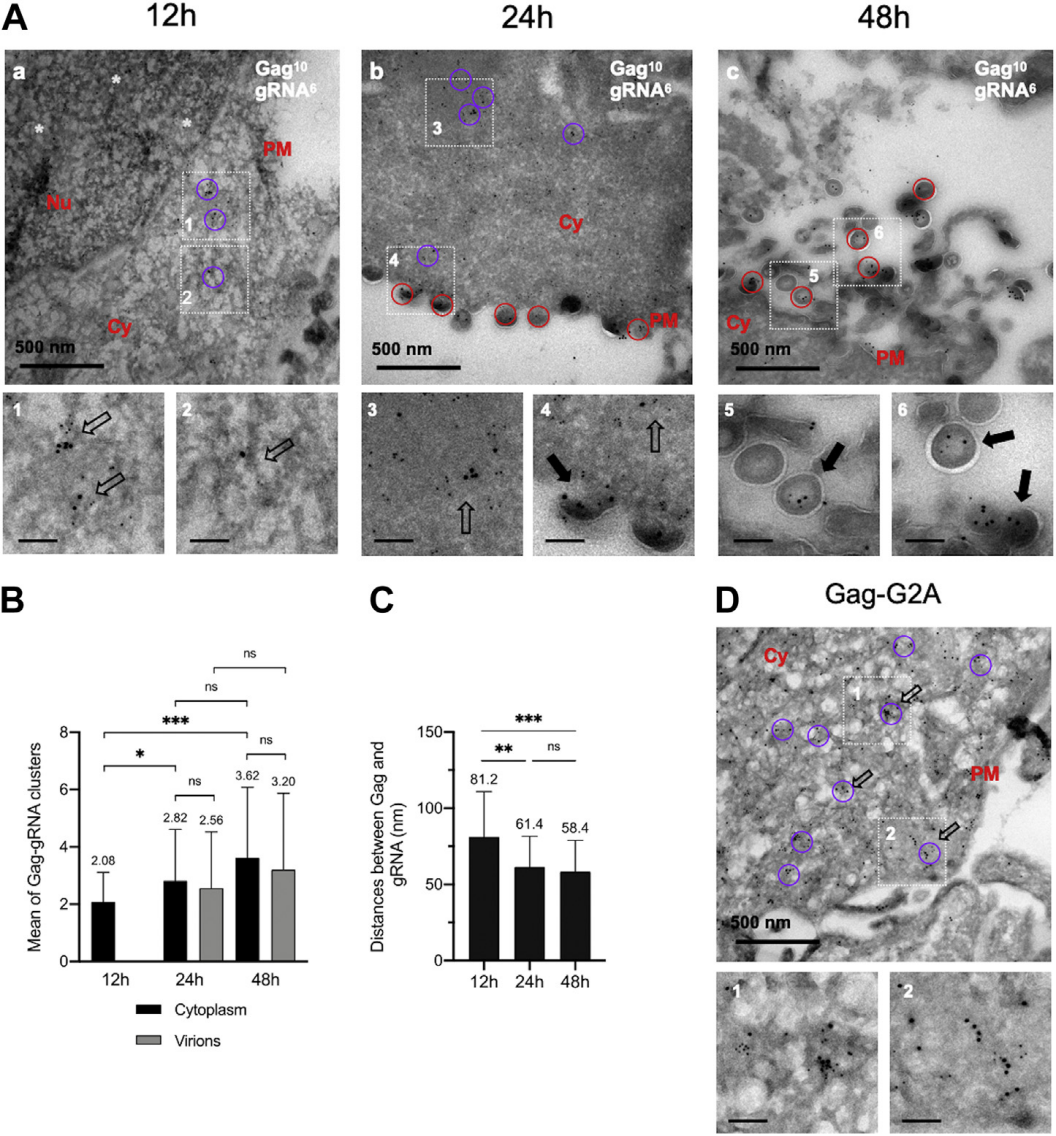
**Dual immunolabeling of HIV-1 Gag and gRNA as function of the time by transmission electron microscopy and morphometric analysis of Gag-gRNA clusters.***A*, HeLa cells were transiently transfected with a mixture of DNA plasmids expressing gRNA-MS2-Δenv and MCP-eGFP-NLS proteins (Ratio 0.6/0.4). After 12 h (a), 24 h (b), or 48 h (c) of expression, the cells were GFP-based sorted, fixed by 4% PFA, embedded in gelatin, and cryo-protected in 2.3 M sucrose. Ultrathin sections were cut and incubated first with a mixture of mouse anti-p24 and rabbit anti-eGFP antibodies, and then with anti-mouse and anti-rabbit antibodies conjugated with 10 nm and 6 nm gold particles, respectively. The circles of 100 to 130 nm in diameters correspond to Gag-gRNA clusters found in the cytoplasm (*magenta*) or in virions (*red*). Insets 1 to 6 show Gag-gRNA clusters, either free in the cytoplasm (*hollow arrow*) or associated with a nascent virion (*full arrow*). The scale is reported at the *bottom left*. Note that at early PT time, Gag-gRNA clusters localized in the cytoplasm, whereas at later, PT Gag-gRNA clusters are found in the virions. *White stars* represent 10 nm gold beads corresponding to Gag in the nucleus. *B*, quantification of Gag-gRNA clusters in cytoplasm and in viruses at each time PT. The data represent the mean ± SEM n = 50 cells from three independent experiments. The *p* values were obtained from Welsch t tests to compare the mean values (ns: nonsignificative, ∗*p* < 0.05, ∗∗∗*p* < 0.0005). *C*, histogram represents the mean distances between Gag and gRNA in each cluster by the measurement of the distance between each 10 nm gold bead and the surrounding 6 nm gold bead at three times PT. The beads distribution has been analyzed bioinformatically. The data shown are the mean ± the SEM from n = 90 cells from three independent experiments. The *p* values were obtained from Welsch t tests to compare the mean distances (ns: nonsignificative, ∗∗*p* < 0.0051, ∗∗∗*p* < 0.0001). *D*, HeLa cells were transiently transfected with a mixture of DNA plasmids expressing gRNA-G2A-MS2-Δenv and MCP-eGFP-NLS protein (Ratio 0.6/0.4) and analyzed, as previously described by TEM. Nu: Nucleus. Cy: Cytoplasm. PM: Plasma Membrane. The scale bar in *black* for zoom magnification corresponds to 100 nm. gRNA, genomic RNA; PFA, paraformaldehyde; PT, posttransfection.

To monitor the time-course of Gag and gRNA localization, HeLa cells were cotransfected with these two plasmids and observed at three times PT (12 h, 24 h, and 48 h) by confocal microscopy (Fig. S2) and by TEM (Fig. 2). By confocal microscopy, both signals were found colocalized in merged images and mostly diffuse in the cytoplasm with an accumulation of Gag at the cell periphery in the form of red dots (Fig. S2). Then, the distribution of Gag and MCP-tagged gRNA was investigated by TEM (Fig. 2). For TEM, Gag was detected by the same anti-p24 antibody whereas the gRNA by anti-eGFP antibody. To ensure the specificity of the observed signals, a fine-tuning for a double labeling on the ultrathin cryo-section was carried out with passivation and extensive washing of the samples. Representative controls are presented in Fig. S1*B*. These electron micrographs showed a tiny amount of 10 nm beads but not of 6 nm beads. None colocalization between 10 nm and 6 nm beads was observed indicating the absence of unspecific signals (Fig. S1*B*).

At 12 h PT, the smaller beads of 6 nm (MCP-eGFP bound to gRNA) were mainly distributed in the nucleus and the cytoplasm, depending on the cell and the expression level (Fig. 2*A*). The larger beads of 10 nm (Gag) were found in the cytoplasm (Fig. 2*A*, a). A few 10 nm beads were also detected in the nucleus (Fig. 2*A*, white stars), which is in agreement with the low nuclear presence of Gag observed by confocal microscopy (Figs. S1*A* and S2) and as previously observed by others (8, 40, 41). Colocalization of at least two beads of 10 nm and two beads of 6 nm was thereafter designed as Gag-gRNA clusters because no such colocalizations were observed in controls. Such clusters occurred essentially in the cytoplasm (Fig. 2*A*, insets 1 and 2, empty arrows) and were rarely observed in the nucleus neither by confocal microscopy nor by TEM (Figs. S2*C* and S5). Only few, if any, virions were observed at the PM in agreement with the low quantity of virus produced at early time PT (42). At 24 h and 48 h PT, the 6 nm beads (gRNA) were found in the nucleus (data not shown), in the cytoplasm and at the level of the PM (Fig. 2*A*, b and c). The 10 nm beads (Gag) were found in the cytoplasm and at the cell periphery where they condensed to form virions (Fig. 2*A*, b and c). At these times, 6 and 10 nm beads were also clustered in the cytoplasm (Fig. 2*A*, inset 3, empty arrow), at the cell periphery (Fig. 2*A*, inset 4, empty arrow), in budding virions (Fig. 2*A*, inset 4, full arrow) or in released virions (Fig. 2*A*, insets 5 and 6, full arrows).

Taken together, these results suggest that the process of Gag and gRNA clustering starts in the cytoplasm once these viral molecules reach the cytoplasm. These clusters then move toward the PM where more Gag are added to complete assembly and form new viral particles.

### Quantitative analysis of Gag-gRNA clusters

We then determined the number of Gag-gRNA clusters and their subcellular distribution in 70 nm-thick sections. Therefore, the scale of the image was used to define a circle of 100 to 130 nm in diameter similar to that of HIV-1 particles (43) (Fig. 2*A*, colored circles). Magenta circles correspond to clusters found in the cytoplasm or near the plasma membrane and red circles represent clusters found in the virions. We then used this footprint as a unit of Gag-gRNA cluster to estimate the number of clusters in each electron micrograph (Fig. 2*B*). For each time PT, we analyzed ∼50 electron micrographs obtained from three independent experiments. At 12 h PT, we counted 104 clusters in the cytoplasm (as described Fig. 2*A*, inset 1), meaning an average of 2.08 ± 1.03 clusters per electron micrograph. At 24 h PT, we detected 269 clusters with an average of 5.38 ± 2.84 clusters per electron micrograph, 2.82 ± 1.79 clusters were in the cytoplasm and 2.56 ± 1.96 in virions (as described in Fig. 2*A*, inset 4). At 48 h PT, we detected 341 clusters with an average of 6.82 ± 3.63 clusters per electron micrograph, 3.62 ± 2.46 clusters were in the cytoplasm and 3.20 ± 2.67 in virions. Note that a significant increase of Gag-gRNA clusters was observed as function of the time. Nevertheless, the ratio of clusters in the cytoplasm over clusters in the virion was similar (compare black and gray boxes of Fig. 2*B*).

We then calculated the distance between Gag and gRNA inside the clusters. Therefore, we reused the circles of 130 nm in diameter to select a mean of 90 ± 10 regions of interest containing Gag and gRNA clusters. The beads were first detected with robust wavelet spot detection (44) and classified according to their size. We then calculated the average distance between 10 nm to 6 nm beads within the circles. We found that the average distance between Gag and gRNA at 12 h, 24 h, and 48 h PT was 81.2 ± 29.7 nm, 61.4 ± 20.2 nm, and 58.4 ± 20.5 nm, respectively (Fig. 2*C* and Table 1). Note that this distance was significantly different between 12 h and 24 h PT, suggesting that Gag-gRNA was condensed as function of the time and then stabilized when the assembly is completed.

**Table 1:** Analysis of HIV-1 Gag-gRNA clusters and gRNA packaging

TEM constant/protein name	Mean distance between Gag and gRNA (nm)	WT *versus* mutant *p* value	Size effect	Percentage of virus containing the gRNA (%)
Gag-WT 12 h	81.2 ± 29.7	/	/	No viruses
Gag-WT 24 h	61.4 ± 20.2	/	/	25
Gag-WT 48 h	58.4 ± 20.5	/	/	40
Gag-ΔNC	93.9 ± 29.6	∗∗∗	L	Viruses without gRNA
Gag-ΔZF1ΔZF2	105.4 ± 35.2	∗∗∗	L	Viruses without gRNA
Gag-F16A	78.4 ± 32.9	∗	M	18
Gag-W37A	98.2 ± 37.6	∗∗∗	L	9
Gag-F16AW37A	90.0 ± 37.1	∗∗∗	L	14
Gag-H23C	85.3 ± 35.4	∗∗∗	L	22
Gag-H44C	85.9 ± 37.2	∗∗∗	L	14
Gag-H23CH44C	83.3 ± 35.0	∗∗∗	L	Few viruses or retained at PM
Gag-G2A	51.7 ± 21.5	Ns	S	No virus
Gag-Δp6	72.8 ± 26.3	Ns	S	No virus
gRNA-ΔSL1	61.8 ± 27.1	Ns	N	36
gRNA-ΔSL1ΔSL3	112.7 ± 39.0	∗∗∗	L	16

**Figure 3:**
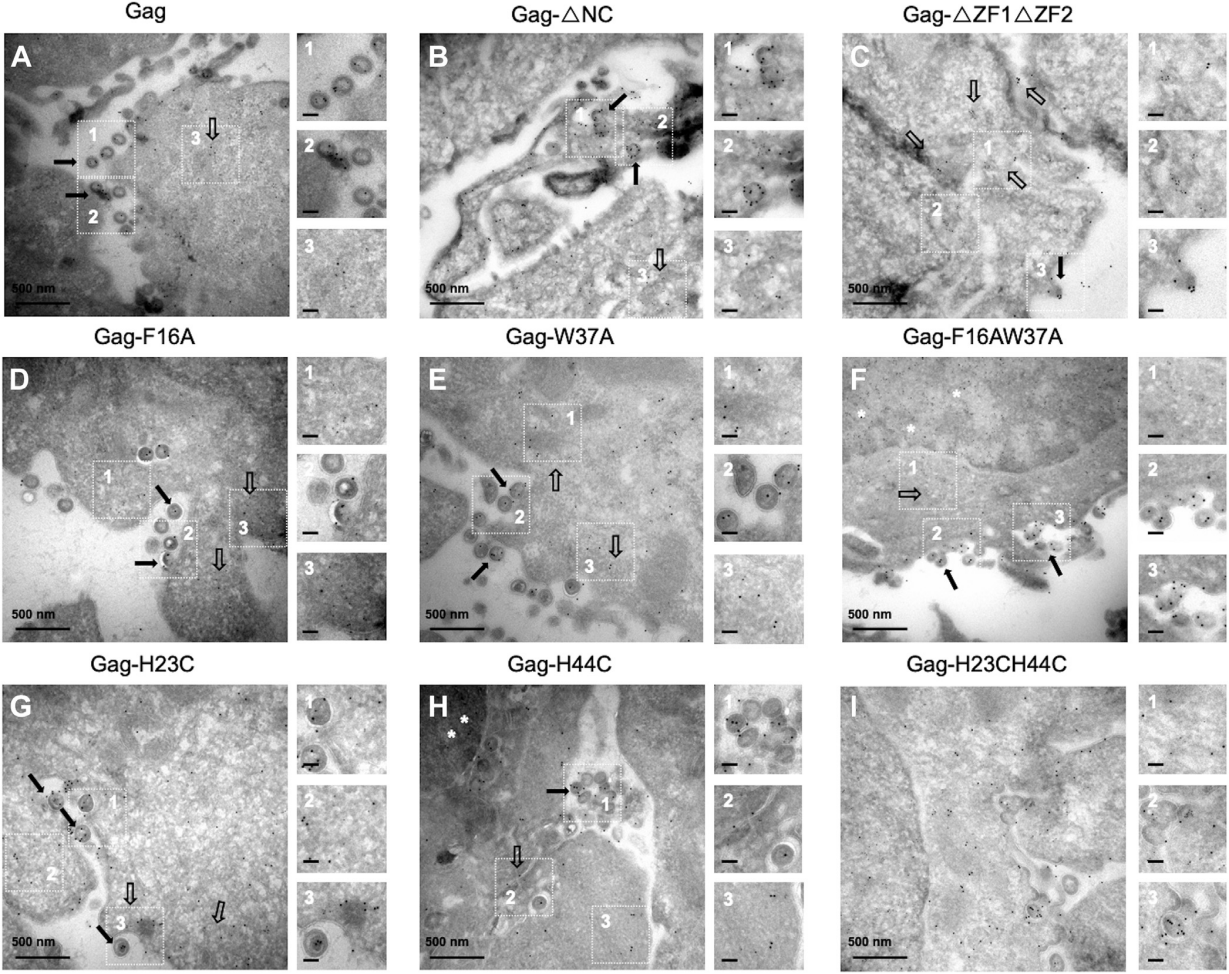
**Impact of NC deletions or ZF punctual mutations on HIV-1 Gag and gRNA clustering, monitored by TEM.** HeLa cells were cotransfected with a mixture of modified pNL4.3-MS2-Δenv encoding for Gag (*A*), Gag-ΔNC (*B*), Gag-ΔZF1ΔZF2 (*C*), Gag-F16A (*D*), Gag-W37A (*E*), Gag-F16AW37A (*F*), Gag-H23C (*G*), Gag-H44C (*H*), or Gag-H23CH44C (*I*) and a plasmid expressing the MCP-eGFP-NLS protein. The cells were observed 24 h PT by TEM. The cells were prepared and stained as presented in Figure 2. Each panel shows the major observed phenotype. For each image, insets 1 to 3 show the clusters of Gag and gRNA-MS2-Δenv either free in the cytoplasm (*hollow arrow*) or associated with a nascent virus-like particle (*full arrow*). *White stars* represent 10 nm gold beads corresponding to Gag in the nucleus. The scale bar in *black* for zoom magnification corresponds to 100 nm. gRNA, genomic RNA; PT, posttransfection; TEM, transmission electronic microscopy; ZF, zinc finger.

**Figure 4:**
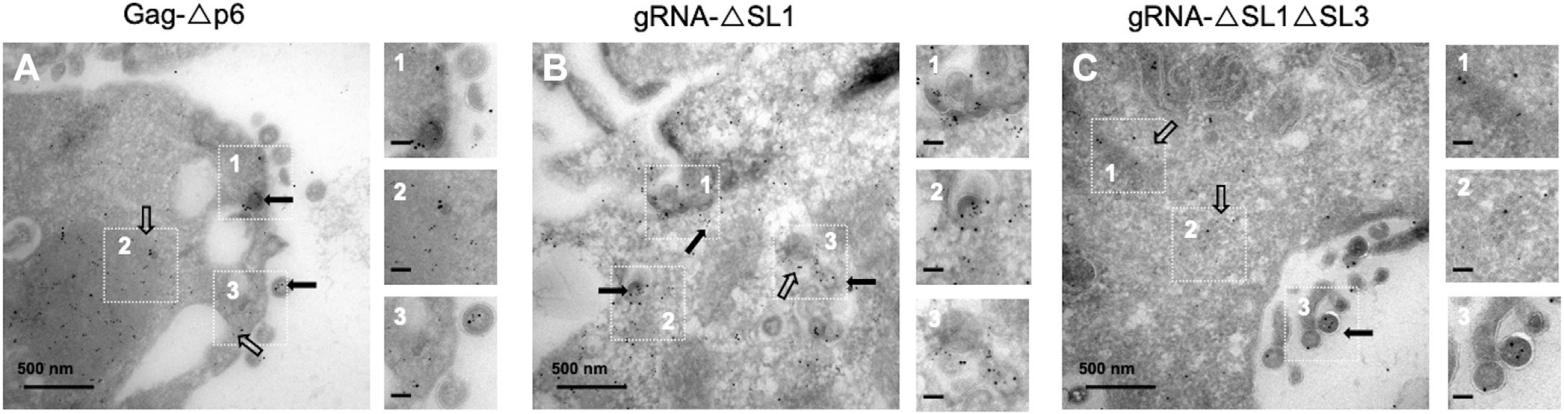
**Impact of HIV-1 Gag or gRNA mutations on Gag-gRNA clustering, monitored by TEM.** HeLa cells were cotransfected with a mixture of modified pNL4.3-MS2-Δenv encoding for Gag-Δp6 (*A*), gRNA-ΔSL1 (*B*), or gRNA-ΔSL1ΔSL3 (*C*) and a plasmid expressing the MCP-eGFP-NLS protein. The cells were prepared and stained as presented in Figure 2. Each panel shows the major observed phenotype. For each image, insets 1 to 3 show the clusters of Gag and gRNA-MS2-Δenv either free in the cytoplasm (*hollow arrow*) or associated with a nascent virus (*full arrow*). The scale bar in *black* for zoom magnification corresponds to 100 nm. gRNA, genomic RNA; TEM, transmission electronic microscopy.

Electron microscopy images of HeLa cells presented in Figure 2, Figure 3, Figure 4 were used to monitor distances between Gag and gRNA. The Table shows the mean distances measured between each 10 nm gold bead and the surrounding 6 nm gold bead in each 100 to 130 nm circle, corresponding to Gag and gRNA respectively. The Table also indicates the percentage of gRNA present in viruses for each condition. The data were obtained from the analysis of 30 and 50 electron micrographs for each analysis respectively, obtained from three independent experiments. The results were statistically analyzed by Welsh *t* test (ns: nonsignificative; *p* > 0.05; ∗*p* < 0.05; ∗∗*p* < 0.005; ∗∗∗*p* < 0.0005) and Cohen d test (N: negligible, d < 0.2; S: small, 0.2 < d < 0.5; M: moderate, 0.5 < d < 0.9; L: large, d > 0.9).

Last, we calculated the ratio of gRNA-containing particles to empty particles. Among the 50 analyzed images obtained at 24 h PT, we counted 515 virions and found that 25% of them were stained with 6 nm beads suggesting that a quarter of virions were filled with gRNA (Table 1). The absence of tagged-gRNA in these particles could be explained by the ability of Gag to self-assemble on cellular RNAs that are not detected in such experimental conditions (2). Another possibility could be that the epitopes of eGFP were not easily accessible as suggested by the few virus-like particle(s) observed without Gag labeling (Fig. S1*A*, insert 2, blue arrow). However, at 48 h PT, among the 50 electron micrographs analyzed, we counted 597 virions. We noted a clear increase in the number of released particles (Fig. 2*A*, compare panels b and c) and 40% of the virions contained the gRNA (Table 1). Thus, the ratio of full to empty virions increased between 24 h and 48 h, suggesting that specific packaging of the gRNA was improved at late times of expression.

### Gag-gRNA clustering does not depend on Gag-membrane binding

To confirm that Gag–gRNA complexes form clusters in the cytoplasm, the glycine residue at position 2 was replaced by an alanine (Gag-G2A; Fig. 1*B*) that abolishes Gag accumulation at the PM and particle budding (45). The production of Gag-G2A in cells was similar to Gag-WT (Fig. S3*A*, gel on the top), whereas as expected, Gag-G2A was not found in the supernatant by ELISA (Fig. S3*B*). We observed that Gag-G2A and gRNA were colocalized in the cytoplasm both by confocal microscopy (Fig. S5*B*) and by TEM with many Gag-gRNA clusters (Fig. 2*D*, insets 1 and 2). The average distance between the two partners was 51.66 nm similar to that observed for Gag-WT (Table 1), indicating that the interaction between Gag and the gRNA occurred in the cytoplasm and did not require Gag anchoring into the PM.

### Aromatic and histidine residues of the NC zinc fingers are important for Gag-gRNA clustering

To investigate by TEM, the mechanism of Gag-gRNA cluster formation, NC, or the zinc fingers were deleted. The deletion of NC or both zinc fingers is detrimental to the Gag–gRNA interaction (11, 12, 15). The expression of the Gag-ΔNC or Gag-ΔZF1ΔZF2 mutant proteins (Fig. 1*B*) were monitored in cells using Western blot at 24 h PT (Fig. S3). The two truncated Gag forms were equally expressed (Fig. S3*A*, gel on the top) even though Gag-ΔNC was processed in contrast to Gag-ΔZF1ΔZF2. Gag-ΔNC was twice less abundant in the supernatant as compared with that of the WT, whereas Gag-ΔZF1ΔZF2 was at a level below 10% (Fig. S3*B*). The reason why Gag-ΔZF1ΔZF2 was not processed remained obscure, but the lack of particles in the supernatant when Gag processing was impaired has already been reported (46).

Then, we examined the expression of truncated Gag and gRNA by confocal microscopy at 24 h PT. In agreement with previous studies, we observed truncated Gag at the PM and diffuse in the cytoplasm, whereas the gRNA was essentially located in the cytoplasm (Fig. S4, *B* and *C*) (11, 12, 15, 34, 36, 37). Next, the cells expressing Gag-ΔNC (Fig. 3*B*, insets 1–3) or Gag-ΔZF1ΔZF2 (Fig. 3*C*, insets 1 and 2) were analyzed by TEM, and Gag-ΔNC or Gag-ΔZF1ΔZF2 and gRNA were found distributed in the cytoplasm at an average distance of 93.9 ± 29.6 nm and 105.4 ± 35.2, respectively for Gag-ΔNC or Gag-ΔZF1ΔZF2 (Table 1). These distances were statistically different from Gag-WT with its cognate gRNA and corresponded to the limit of what we defined as a cluster. Note that in agreement with the ELISA monitoring (Fig. S3*B*) some free empty virions were observed for Gag-ΔNC (Fig. 3*B*, insets 1 and 2) but not for Gag-ΔZF1ΔZF2 (see inset 3 of Fig. 3*C* membrane protrusion) (15, 37). These results confirmed that NC and both zinc fingers are essential for Gag-mediated recruitment of gRNA in the cytoplasm.

We then used NMR data of the complex between NCp7 and oligonucleotides to pinpoint NC residues involved in the specific recognition of the gRNA. The results showed that F16 and W37 residues located in the proximal and the distal zinc finger, respectively, were in a close vicinity in the complex and involved in stacking interactions with the nucleotide bases (27, 29, 47, 48). Mutated pNL4.3-MS2-Δenv plasmids with either the F16A, W37A or both F16AW37A substitution in GagNC domain were constructed (Fig. 1*B*). The Gag mutants were equally expressed in cells as compared with WT Gag (Fig. S3*A*, lower gel). We also noticed that the F16A mutation decreased the amount of Gag produced in the supernatant by 40%, whereas the W37A mutation had no effect (Fig. S3*B*). As a consequence, the amount of double mutant in the supernatant is reduced by 25%. The detection of Gag mutants by confocal microscopy showed Gag diffuse in the cytoplasm and accumulated at the PM (Fig. S4, *D*–*F*). By TEM, Gag-F16A (Fig. 3*D*), Gag-W37A (Fig. 3*E*), and the double mutant Gag-F16AW37A (Fig. 3*F*) were found in the cytoplasm and assembled in viral particles at the PM. A careful analysis of the mutant virions revealed that the ratio of particles containing gRNA to deficient particles dropped down to 18% for Gag-F16A and 9% for Gag-W37A virions, which was significantly lower than Gag-WT (25%) (Table 1). The mean distances between the 6 and 10 nm beads were 78.4 nm, 98.2 nm, and 90.0 nm for Gag-F16A, Gag-W37A, and Gag-F16AW37A with their cognate gRNA, respectively (Table 1). Note that the Gag-gRNA distance for Gag-F16A was only moderately increased as compare with WT-Gag (*p* < 0.05, M) in contrast to that for Gag-W37A (*p* < 0.0005, L), suggesting that the two aromatic residues are not equivalent in Gag–gRNA complex.

To examine the influence of the zinc finger structure on Gag-gRNA clustering, we generated two Gag derivatives where the zinc ligands, histidines 23 and 44, were changed to cysteine (Fig. 1*B*). These substitutions modify the NC structure but only slightly the binding of zinc ion (49). These H/C substitutions have a minor effect on *in vitro* NC-RNA interaction, chaperone activity of NC (50, 51, 52), and viral DNA synthesis (53, 54, 55) but have a drastic impact on gRNA packaging (54) and virus infectivity (15, 23, 56). Western blot analysis showed that Gag-H23C, Gag-H44C, and Gag-H23CH44C were equally expressed and processed by the viral protease as WT Gag. Nevertheless, as seen by ELISA, a significant decrease of virions in the supernatant was observed for all these constructs (Fig. S3*B*). By confocal microscopy, we saw no difference in the staining of Gag derivatives and cognate gRNA compared with what was observed in WT condition, so that both partners diffused into the cytoplasm and protein accumulated at the PM level (Fig. S4, *G*–*I*). However, by TEM, mutated Gag-H23C, Gag-H44C, and Gag-H23CH44C appeared diffuse in the cytoplasm (Fig. 3, third row). For Gag-H23C and Gag-H44C, budding particles were observed (Fig. 3*G*, insets 1 and 3 and Fig. 3*H*, insets 1 and 2), whereas only few particles were observed for Gag-H23CH44C, most of them being tethered to the PM (Fig. 3*I*, insets 2 and 3). In addition, these protein mutants were poorly clustered with the gRNA with a mean distance of 85.3 nm, 85.9 nm, and 83.2 for Gag-H23C, Gag-H44C, and GagH23CH44C, respectively (Table 1). The level of gRNA packaging in virions was 22% and 14% for Gag-H23C and Gag-H44C, respectively (Table 1).

Taken together, these results show that mutating a single residue in either the first or second zinc finger has an impact on the formation of the Gag–gRNA complexes. Furthermore, point mutations in the distal zinc finger (W37 or H44) have a more pronounced effect on the distance between Gag and the gRNA and on the ratio of full to empty particles than do similar point mutations in the proximal zinc finger.

### The p6 domain is dispensable for Gag-gRNA clustering

NMR studies and RNA-binding experiments on NCp15 (NCp7, p2, and p6) have shown that p2 and p6 domains, while they are not directly involved in RNA, could modulate the binding properties of NC domain with nucleic acid (49, 57, 58). Also recent biochemical and biophysical experiments on Gag-Δp6 proposed a role of the p6 domain in the specificity of Gag-gRNA recognition and interaction (59). More recently, the group of Musier–Forsyth showed that Gag-Δp6 tightly interacts with the viral Psi RNA packaging signal (60). This prompted us to study the contribution of a Gag deleted of the p6 domain (Gag-Δp6) to Gag-gRNA clustering. By Western blot, we found that Gag-Δp6 was less produced (Fig. S3*A*, upper gel) than Gag-WT. The protein released in the medium of transfected cells was examined by ELISA and as expected, a strong decrease down to 9% of the particles was found upon harvest (Fig. S3*B*) (60, 61). By confocal microscopy, we observed an important colocalization of Gag-Δp6 and gRNA (Fig. S5*C*) and by TEM, complete particles sticking to the PM were found (Fig. 4*A*, inset 3) in agreement with the fact that Gag-Δp6 does not recruit the ESCRT machinery necessary for the release of the viral particles from the PM (62, 63). We observed many Gag-Δp6-gRNA clusters in the cytoplasm (Fig. 4*A*, insets 1 and 2) and in stuck virions (Fig. 4*A*, inset 3). The mean distance between the 6 and 10 nm gold beads was 72.8 nm similar to that observed with WT clusters (Table 1). It can be concluded that the interaction of Gag with the gRNA is independent from the p6 domain of Gag.

### HIV-1 gRNA association with Gag involves an RNA domain larger than SL1

Virus assembly upon gRNA packaging requires specific interactions between Gag and the packaging signal Psi located in the 5′ UTR of the gRNA. Psi is thought to encompass the stem loops (SLs) SL1, SL2, and SL3 overlapping the RNA dimerization sequence (18, 64). SL1 is the dimer initiation signal corresponding to a GC-rich sequence and adopts a kissing loop structure that facilitates the gRNA dimerization and the specificity of gRNA packaging (39, 65, 66, 67, 68, 69). *In vitro* studies have recently shown that the SL1 stem-loop was also the primary binding site for Gag (25), which prompted us to investigate the role of SL1 in gRNA packaging in our model system. First, we showed that SL1 deletion in pNL4.3-MS2-Δenv plasmid (Fig. 1*A*, first stem-loop in pink) had no impact on the level of cytoplasmic Gag and of viral particles released in the supernatant (Fig. S3*B*). Moreover, we observed by confocal microscopy that SL1 deletion had no impact on gRNA nuclear export as evidenced by a high level of gRNA imaged in the cytoplasm. In addition, we observed an important colocalization of WT Gag and gRNA-ΔSL1 (Fig. S5*D*). TEM analyses confirmed the presence of Gag-gRNA-ΔSL1 clusters in the cytoplasm and close to the PM (Fig. 4*B*, inset 1). A third of viral particles contained Gag and gRNA-ΔSL1 (Fig. 4*B*, insets 1 and 2), and the average distance between the beads was 61.8 nm, similar to that for WT particles (Table 1).

Next, the downstream SL3 structure known to tightly interact with NC (27) and to contain nucleotide sequences essential for specific genome packaging (27, 28, 70, 71, 72) was deleted. The plasmid pNL4.3-ΔSL1ΔSL3-MS2-Δenv was constructed (Fig. 1*A*, stem-loop in pink) and after transfection in HeLa cells, Gag was found both in the cell and in the supernatant at a level similar to that for the WT (Fig. S3*A*, upper gel and Fig. S3*B*). The expression of Gag and gRNA-ΔSL1ΔSL3 was also monitored by confocal microscopy showing a diffuse pattern in the cytoplasm and at the cell periphery (Fig. S5*E*). In the cytoplasm, few clusters of Gag-gRNA-ΔSL1ΔSL3 were observed by TEM (Fig. 4*C*, insets 1 and 2), and the average distance between Gag and gRNA-ΔSL1ΔSL3 was increased to 112.7 nm (Table 1). We observed a consequent number of particles, in agreement with ELISA, and 16% of them contained gRNA-ΔSL1ΔSL3 as compared with the 25% of WT gRNA packaged (Fig. 4*C*, inset 3 and Table 1).

## Discussion

According to previous studies using HeLa or Jurkat cells, the initial interaction between HIV-1 Gag and gRNA kick-start assembly that take place in the cytoplasm (4, 8, 11, 12, 39, 73) or at the PM (7, 39, 74, 75). Here, we investigated the formation of the Gag-gRNA clusters in HeLa cells at higher resolution by quantitative TEM at 12 h, 24 h, and 48 h PT. This approach allows the observation of Gag-gRNA clusters and ultrastructural details of their cellular environment in cells producing the virus particles. The observation of more than 500 clusters confirm that the formation of the nucleoprotein complexes starts in the cytoplasm and accumulate at the PM. In fact, at 12 h PT, Gag and the gRNA formed clusters close to the nuclear envelope and in the cytoplasm suggesting that the gRNA associates with Gag most probably soon after its synthesis and nuclear export. At 24 h and 48 h PT, an increase of Gag-gRNA clusters was observed in the vicinity of the PM. We cannot completely exclude that the Gag–gRNA interactions occur directly at the PM as observed by Total Internal Reflection Fluorescence microscopy (7, 76), but such clusters were hardly seen 12 h PT. Other works using confocal or more quantitative imaging approaches have also shown that the HIV Gag–RNA interaction was cytoplasmic and at the PM (11, 12, 15, 73, 77) and for FIV at the rim of the nucleus (8). Nevertheless, the high resolution provided by TEM allows to distinguish nucleoprotein complexes in the cytoplasm from those incorporated in the virus particles.

Note that 10 to 20% of Gag was found diffusing in the nucleus as seen by confocal microscopy (Figs. S1–S4) and TEM (Fig. 2). In agreement with the work of Parent’s group (41), only few Gag-gRNA clusters were found in the nucleus by confocal microscopy (Figs. S2 and S5), rendering their difficult observation by TEM. Thus, it cannot be excluded that HIV Gag–gRNA interaction takes place in the nucleus, but this seems to concern very few vRNPs. In contrast, the nuclear trafficking of RSV Gag is more documented, and it has been recently shown by FRET and BiFC that RSV Gag interacts with gRNA inside the nucleus (78). This nuclear interaction was proposed to be essential for RNA packaging in virions even though a poor impact on virus replication was observed with a RSV Gag construct deficient for nuclear trafficking (79).

At 24 h PT, about 25% of the particles contain the gRNA and 40% at 48 h PT. This increase over time of the Gag-gRNA ratio is probably related to the time-dependent increase in the concentration of the two partners (7, 74). However, in agreement to the results obtained with MLV where 20% of particles were found containing viral RNA (80), these results indicate that the majority of the HIV viral particles lacks gRNA. These particles are not empty because they contain cellular RNAs (30, 33, 71, 81). Nevertheless, the low proportion of gRNA-containing particles could also result from eGFP epitopes not detected by anti-eGFP or out of the 70 nm width of the section. Indeed, previous studies showed that 75 to 90% of the HIV-1 particles contain the Psi RNA (7, 82). But in the report of Jouvenet *et al.*, Gag/Gag-mCherry were expressed *in trans* under the control of cytomegalovirus promoter that produces a large amount of protein which in turn promotes the RNA packaging, whereas in Chen *et al.* (82), Gag-CFP was expressed in *cis* but in 293T cells known for their ability to produce very large amount of foreign protein upon DNA transfection. Last, the discrepancy can also be attributed to the limited resolution of the optical microscopy that is a major obstacle for the observation of individual viral particles with or without gRNA, whereas TEM resolution allows to discriminate empty from full particles. Whatever the reasons for these differences, it is likely that this limit will be independent of the different mutations, which allowed us to compare our different Gag constructions for their property to form clusters and to package the gRNA.

An original aspect of our approach is that we were able to provide quantitative data by processing TEM images. For this purpose, we developed a bead detection model for TEM images built upon a method initially used for robust spot detection in fluorescence microscopy (44), and this image processing approach was implemented on Matlab. This allowed to semi-automatically evaluate the distance between Gag and the gRNA as function of time and of the type of mutants. In the Gag-gRNA clusters, the distance between the two components was ∼ 60 nm at 24 h PT in agreement with the size of the particles. To further validate this distance and to estimate the distribution of each partner, we used Gag-G2A that was previously found to tightly interact with the gRNA (11, 12) and a value of 51.7 ± 21 nm was found (Fig. 2*D*). Interestingly, the mean distance between Gag and gRNA decreased between 12 h and 48 h PT (Fig. 2*C* and Table 1), suggesting that during the nucleoprotein transport in the cytoplasm to reach the PM, the Gag-gRNA clusters tend to undergo a condensation phenomenon.

However, a careful study (Table 1) shows that it is difficult to correlate the distance between Gag and gRNA with gRNA packaging. Knowing that all mutants are produced at a similar level (Fig. S3*A*), we can hypothesize that this distance is proportional to the affinity between the two partners. The zinc finger motifs of NCp7 being very important for the Gag–gRNA interaction, their deletion/mutation results in an increase of this distance. Similarly, the deletion of several LTR domain motifs also results in an increase in this Gag-RNA distance. Conversely, the mutation of other residues such as G2A and p6 that are not directly involved in the Gag-gRNA interaction had no effect on this distance. In line with this, the encapsidation of gRNA depends on other factors such as the assembly of Gag, its intracellular trafficking, and its attachment to the PM (18, 64). In addition, we noted that point mutations in the first finger (F16A and H23C) caused a small increase of the distance to ∼80 nm and at the same time, gRNA-containing particles slight decrease as compared with that obtained for the WT Gag protein. Similar point mutations were performed in the second zinc finger (W37A and H44C) causing a larger distance between Gag and the gRNA to be around ∼90 to 95 nm accompanied with a larger drop of packaged gRNA in virion pellets. Taken together, the two mutations in the first finger appear to have a smaller effect than similar mutations in the second finger and confirms that both zinc fingers do not have the same function during the assembly (11, 23, 83, 84).

The relative importance of the proximal finger of the NC as compared with the distal in RNA recognition is clearly supported by recent NMR data showing that ZF2 is more prone to interact with RNA than ZF1 (85, 86). It should be noted that these point mutations in each ZF do not have as great an effect as the deletion of the GagNC (Fig. 3). Indeed, if the structure of the zinc finger motifs directs the intercalation of these two aromatic residues in the nucleic bases, the spatial proximity of the dactyl motifs in NCp7 allows the formation of a large hydrophobic plateau including V13, F16, I24, A25 in ZF1 and W37, and G45 and M46 in ZF2 that reinforces this GagNC–RNA interaction (27, 29). Thus, the loss of the two aromatic residues would be partially compensated by the presence of other residues of the hydrophobic plateau.

Recent study showed that the p6 domain of Gag was important to discriminate specific from nonspecific RNAs *in vitro* (59), whereas another one indicated that Gag and Gag-Δp6 interact similarly with Psi-containing RNA and promoting gRNA packaging (60). A careful analysis of cells expressing Gag-Δp6 revealed large amount of clusters composed by Gag-Δp6 and the gRNA with a mean distance of 72.8 nm. As anticipated, these clusters were only found in the cytoplasm in agreement with the role of p6 in virus budding (Fig. 4*A*) (63). These findings indicate that the p6 domain is most probably not involved in the specific interaction between Gag and the gRNA.

How is the gRNA recognized by Gag? This has been extensively studied showing that SLs located in the large 5′UTR play a critical role in RNA packaging (87). In the present study, we show by TEM the presence of numerous Gag-gRNA-ΔSL1 clusters (Fig. 4*B*), with an average distance between the two partners and a ratio of empty to full particles identical to what was found for WT Gag-gRNA. Even though the data provided by TEM cannot quantify the affinity between Gag and gRNA-ΔSL1, this result suggests that SL1 is not the major *cis*-acting factor for RNA packaging. In fact, the deletion of SL1 or mutations in the SL1 dimer initiation signal were shown to leave the genome packaging unaffected, whereas others showed that such mutations cause a moderate decrease of genomic RNA packaging (67, 71, 88, 89, 90, 91). A specific interaction between Gag and the SL1 stem-loop was found *in vitro* (25), but in our work it may be hypothesized that, in cellular context, the SL1 deletion has been rescued by the presence of the other SL of the 5′LTR such as SL3 (87, 92).

In line with this, the deletion of SL3 together with SL1 caused a significant decrease of Gag-gRNA clusters with an increase of Gag-RNA distance and a drop of packaged gRNA (Table 1 and Fig. 4*C*). These data are in agreement with a series of reports showing that SL3 contributes to the RNA packaging (28, 71, 93) and of the fact that GagNC tightly binds (27, 92). Nevertheless, here again, these two deletions did not completely abrogated packaging of ΔSL1ΔSL3gRNA in nascent particles that could be interpreted by the fact that Gag can interact with multiple sequences in addition to SL1 and SL3, notably SL2 (48) and to several distinct stretches located in the UTR (70, 87, 92) and in the RRE domain (94, 95).

In conclusion, the data presented sheds new light on HIV-1 assembly. They confirm that in HeLa cells, Gag molecules may initially interact with the gRNA in the cytoplasm. Using a method to calculate the distance between Gag and the gRNA, we show that during Gag–gRNA complex trafficking to the PM, a condensation process takes place, most probably in a membrane-less mode directed by NC (96). Moreover, packaging of the gRNA is far from being efficient possibly because of a nonspecific RNA-binding activity of GagNC. At least if both zinc fingers are essential in Gag for gRNA packaging, they are not fully equivalent.

Overall, our original approach based on morphometric analysis of gold beads in TEM coupled to quantify Gag-gRNA colocalization could be used to study other viral assembly models or more generally RNA/protein interactions.

## Experimental procedures

### Plasmid DNA

Plasmids pNL4.3-MS2-Δenv, pNL4.3-ΔZF1ΔZF2-MS2-Δenv, and pNL4.3-ΔSL1-MS2-Δenv, containing 24 repetitions of MS2 stem-loop in the pol gene of the NL4.3 backbone, were described (39). The pNL4.3-ΔNC-MS2-Δenv mutant was generated from pNL4.3-MS2-Δenv plasmid by deletion of the NC domain using PCR-based deletion mutagenesis with forward primer 5′-ACCATAATGCAGGCTAATTTTTTAGGGAAGATC-3′ and reverse primer 5′-TAAAAAATTAGCCTGCATTATGGTAGCTGGATTTGTTAC-3′. The pNL4.3-H23C-MS2-Δenv and pNL4.3-H44C-MS2-Δenv were constructed using PCR site-directed mutagenesis with the forward primer 5′-AAAGAAGGGTGCATAGCCAAAAATTGC-3′ and the reverse primer 5′-GCCACAATTGAAACACTTAACAGTC-3′ for H23C and forward primer 5′-AGGAAGGATGCCAAATGAAAGATTGTACTG-3′ and reverse primer 5′-TTCCACATTTCCAACAGCCCTT-3′ for H44C. The pNL4.3-F16A-MS2-Δenv and pNL4.3 W37A-MS2-Δenv were constructed using PCR site-directed mutagenesis with forward primer 5′-TGTTAAGTGTGCCAATTGTGGCAA-3′ and reverse primer 5′-GTCTTTCTTTGGTTCCTAAAATTG-3′ for F16A and forward primer 5′- AAAGGGCTGTGCGAAATGTGGAAAGGAAGG-3′ and reverse primer 5′- TTCCTAGGGGCCCTGCAAT-3′ for W37A. The pNL4.3-G2A-MS2-Δenv derivate was constructed using PCR site-directed mutagenesis with forward primer 5′-AGAGAGATGGCTGCGAGAGC-3′ and reverse primer 5′-CCTTCTAGCCTCCGCTAGTCA-3′. The pNL4.3-Δp6-MS2-Δenv was constructed by inducing a stop codon in p6 domain using forward primer 5′-TTCTTTAGAGCTGACCATAGCCAACAG-3′ and reverse primer 5′- AATTCCCTGGCCTTCCCTTGTG-3′. The pNL4-3-ΔSL1ΔSL3-MS2-Δenv derivate was generated from pNL4-3-ΔSL1-MS2-Δenv plasmid by deletion of the SL3 stem-loop using PCR-based deletion mutagenesis using forward primer 5′-AAGGAGAGAGATGGGTGCGAGAGCGTCG-3′ and reverse primer 5′-TCAAAATTTTTGGCGTACTCACCAGTCGCC-3′.

The MCP-eGFP-NLS sequence was obtained from Querido and Chartrand (97) and included in a pCDNA3.1(+) DNA backbone using NheI and HindIII restriction sites. The integrity of all plasmid constructs was assessed by DNA sequencing.

### Cell culture and DNA transfection

HeLa cells were grown at 37 °C/5% CO_2_ in Dublecco’s modified Eagle medium (DMEM) supplemented with 10% fetal calf serum (Gibco) and 1% antibiotic mixture (Invitrogen Corporation Pontoise). HeLa cells were transfected with a mixture of pNL4.3-MS2-Δenv plasmid or derivatives with MCP-eGFP-NLS plasmid (ratio 0.6/0.4) by incubating cells with 2 μg of DNA plasmids for confocal microscopy or 10 μg of plasmid for TEM with jetPEI (Life Technologies).

### Western blot

HeLa cells were washed 24 h posttransfection with PBS and resuspended in ice-cold RIPA lysis buffer (50 mM Tris-HCl pH 7.4, 150 mM NaCl, 1% NP40, 0.15% sodium deoxycholate, 1 mM EDTA, and 0.05% SDS supplemented with a complete anti-protease cocktail from Thermofisher). 50 μg of total proteins whom concentrations have been evaluated using Pierce BCA Protein Assay Kit (23227, Thermo Scientific) were heat denaturated, loaded on a 12% SDS-PAGE, and transferred onto polyvinylidene fluoride membrane. Membranes blocked by nonfat milk were probed by a monoclonal anti-p24 antibody (NIH AIDS Reagent, ref 6521) and revealed by enhanced chemiluminescence with horseradish peroxidase-conjugated secondary antibodies on an ImageQuant LAS500 apparatus (GE Healthcare). The protein levels were standardized against GAPDH (Bio-techne, ref NB100-56875).

Virus pellets were obtained by ultracentrifugation through a 20% sucrose cushion (AH-629 rotor for 1h30 at 28,000 rpm) and resuspended in 1× PBS. The p24 concentration was determined by ELISA using Innotest HIV Antigen mAb kit (Fujirebio).

### Confocal microscopy

HeLa cells were fixed by 4% paraformaldehyde and permeabilized with 0.2% X100 Triton/1× PBS for 20 min. The cells were blocked for 30 min with 0.2% bovine serum albumin in 1× PBS and incubated with anti-p24 Gag antibody (NIH AIDS Reagent, ref 6521) and then with Alexa Fluor 594 goat-anti-mouse antibody (ThermoFisher Scientific). DNA was stained with DAPI (2-(4-Amidinophenyl)-6-indolecarbamidine dihydrochloride, SIGMA) (1 mg/ml, 1:10,000). Fluorescence confocal images were taken using a confocal microscope LEICA SP8 gSTED equipped with 60× PL APO 1.30 CS2 Oil and laser diode at 405 nm for DAPI, argon laser at 488 nm for Alexa 488, and white laser at 594 nm for Alexa 594.

### Cell sorting by FACS

Transfected HeLa cells were treated with trypsin, resuspended in ice-cold complete medium (DMEM, 10% fetal bovine serum), and then sorted into 5 ml tubes. A gating was done using the BD FACS ChorusTM software (BD Biosciences). Cell sorting was performed with a 100 μm nozzle and sorted directly into 1.5 ml tubes containing complete medium to minimize cell stress. After establishing the basic gating parameters, GFP positive cells (3.10^5^–1.10^6^) of each population of interest were sorted at a speed of 6000 cells/s.

### Immuno-electron microscopy according to the Tokuyasu method

HeLa cells expressing pNL4.3-MS2-Δenv or derivate plasmids for 24 h were sorted and fixed for 2 h at 4 °C with 4% paraformaldehyde/0.1% glutaraldehyde in Sorensen phosphate buffer (pH 7.6), washed twice with PBS (pH 7.6), and included in 12% gelatin. Infusion with 2.3 M sucrose was performed overnight at 4 °C before ultrathin cryo-sectioning at −100 °C on a Leica Microsystems FC7 cryo-ultramicrotome. Sections of 70 nm thickness were retrieved with a 2% methylcellulose/2.3 M sucrose mixture (1:1) and collected onto formvar/carbon-coated nickel grids.

### Immunogold labeling on ultrathin cryosections

After removal of gelatin at 37 °C, the sections were saturated with bovine serum albumin (BSA) (Aurion) for 30 min at room temperature. After washing in PBS-0.1% BSA (three washes of 5 min each), the sections were incubated with PBS-0.1% BSA containing 1:400 Mouse HIV-1 anti-p24 monoclonal antibody (NIH AIDS Reagent, ref 6521) and 1:200 Rabbit anti-eGFP monoclonal antibody (Abcam, ab6556). After six washes of PBS-0.1% BSA (5 min each), the grids were incubated with PBS-0.1% BSA containing 1:60 gold-conjugated Goat anti-Mouse (10 nm) and 1:50 gold-conjugated Goat anti-Rabbit (6 nm) (Aurion). The grids were finally washed six times in PBS-0.1% BSA (5 min each), six times in PBS (5 min each), and rinsed three times with distilled water. The contrasting step was performed by incubating grids in a 2% uranyl acetate/2% methylcellulose mixture (1:10) for 10 min at room temperature. The sections were imaged with a transmission electron microscope at 100 kV (JEOL 1011).

### Measurement of distances between gold beads in TEM

The picture’s dynamic has been modified nonlinearly to improve beads contrast. The method is based on a “à trou” wavelet decomposition of the picture followed by thresholding of nonsignificant coefficients and multi-scale correlation. This approach allows a bead detection while being more robust to noise and local textures. Noncircular spots have been eliminated, then bead surfaces have been converted in their equivalent diameter, and separated between two classes considering the pixel size and after analyzing their distribution. For each 10 nm bead, we calculated the minimal distance to every 6 nm surrounding bead. Then, we averaged these minimal distances to characterize the relative spatial distribution of the beads.

### Statistical analysis

Statistical analyses were performed using R Studio software. The results are expressed as mean ± SD obtained from the images of three independent experiments. In each experiment, we compared mutant as follows: Welch’s ANOVA was used to make the overall comparison of mutant’s distributions. When the ANOVA suggested a significant difference between groups, we undertook pairwise comparisons using the Games–Howell procedure to control the type I error rate. These two procedures were chosen because of the substantial heterogeneity of variances between the groups. For all the analyses, Welsh *t* test was performed and all *p* values under an alpha risk of 0.05 were considered as significant. We evaluated the magnitude of differences between groups using Cohen’s d test. Confidence intervals of these effect sizes were estimated using the 5th and 95th percentiles of their bootstrap distribution.

## Data availability

The authors declare that the data supporting the findings of this study are available within the article and its supporting information.

## Supporting information

This article contains supporting information (98, 99).

## Conflict of interest

The authors declare that they have no conflicts of interest with the contents of this article.

## References

[1] Sundquist W.I., Krausslich H.-G. (2012). HIV-1 assembly, budding, and maturation. Cold Spring Harb. Perspect. Med..

[2] Comas-Garcia M., Davis S., Rein A. (2016). On the selective packaging of genomic RNA by HIV-1. Viruses.

[3] Ono A. (2009). HIV-1 assembly at the plasma membrane: Gag trafficking and localization. Future Virol..

[4] Kutluay S.B., Bieniasz P.D. (2010). Analysis of the initiating events in HIV-1 particle assembly and genome packaging. PLoS Pathog..

[5] Chen J., Grunwald D., Sardo L., Galli A., Plisov S., Nikolaitchik O.A., Chen D., Lockett S., Larson D.R., Pathak V.K., Hu W.-S. (2014). Cytoplasmic HIV-1 RNA is mainly transported by diffusion in the presence or absence of Gag protein. Proc. Natl. Acad. Sci. U. S. A..

[6] Grigorov B., Arcanger F., Roingeard P., Darlix J.-L., Muriaux D. (2006). Assembly of infectious HIV-1 in human epithelial and T-lymphoblastic cell lines. J. Mol. Biol..

[7] Jouvenet N., Simon S.M., Bieniasz P.D. (2009). Imaging the interaction of HIV-1 genomes and Gag during assembly of individual viral particles. Proc. Natl. Acad. Sci. U. S. A..

[8] Kemler I., Meehan A., Poeschla E.M. (2010). Live-cell coimaging of the genomic RNAs and Gag proteins of two lentiviruses. J. Virol..

[9] Molle D., Segura-Morales C., Camus G., Berlioz-Torrent C., Kjems J., Basyuk E., Bertrand E. (2009). Endosomal trafficking of HIV-1 Gag and genomic RNAs regulates viral egress. J. Biol. Chem..

[10] Perlman M., Resh M.D. (2006). Identification of an intracellular trafficking and assembly pathway for HIV-1 Gag: HIV-1 Gag trafficking pathway. Traffic.

[11] Boutant E., Bonzi J., Anton H., Nasim M.B., Cathagne R., Réal E., Dujardin D., Carl P., Didier P., Paillart J.-C., Marquet R., Mély Y., de Rocquigny H., Bernacchi S. (2020). Zinc fingers in HIV-1 Gag precursor are not equivalent for gRNA recruitment at the plasma membrane. Biophys. J..

[12] Hendrix J., Baumgärtel V., Schrimpf W., Ivanchenko S., Digman M.A., Gratton E., Kräusslich H.-G., Müller B., Lamb D.C. (2015). Live-cell observation of cytosolic HIV-1 assembly onset reveals RNA-interacting Gag oligomers. J. Cell Biol..

[13] Becker J.T., Sherer N.M. (2017). Subcellular localization of HIV-1 Gag-pol mRNAs regulates sites of virion assembly. J. Virol..

[14] Booth A.M., Fang Y., Fallon J.K., Yang J.-M., Hildreth J.E.K., Gould S.J. (2006). Exosomes and HIV Gag bud from endosome-like domains of the T cell plasma membrane. J. Cell Biol..

[15] Grigorov B., Décimo D., Smagulova F., Péchoux C., Mougel M., Muriaux D., Darlix J.-L. (2007). Intracellular HIV-1 Gag localization is impaired by mutations in the nucleocapsid zinc fingers. Retrovirology.

[16] Sherer N.M., Lehmann M.J., Jimenez-Soto L.F., Ingmundson A., Horner S.M., Cicchetti G., Allen P.G., Pypaert M., Cunningham J.M., Mothes W. (2003). Visualization of retroviral replication in living cells reveals budding into multivesicular bodies: Retroviral budding. Traffic.

[17] Parent L.J. (2011). New insights into the nuclear localization of retroviral Gag proteins. Nucleus.

[18] Kuzembayeva M., Dilley K., Sardo L., Hu W.-S. (2014). Life of psi: How full-length HIV-1 RNAs become packaged genomes in the viral particles. Virology.

[19] Mely Y., Cornllle F., Fournié-Zaluski M.-C., Darlix J.-L., Roques B.F., Gérard D. (1991). Investigation of zinc-binding affinities of moloney murine leukemia virus nucleocapsid protein and its related zinc finger and modified peptides. Biopolymers.

[20] Morellet N., Jullian N., De Rocquigny H., Maigret B., Darlix J.L., Roques B.P. (1992). Determination of the structure of the nucleocapsid protein NCp7 from the human immunodeficiency virus type 1 by 1H NMR. EMBO J..

[21] Summers M.F., Henderson L.E., Chance M.R., South T.L., Blake P.R., Perez-Alvarado G., Bess J.W., Sowder R.C., Arthur L.O., Sagi I., Hare D.R. (1992). Nucleocapsid zinc fingers detected in retroviruses: EXAFS studies of intact viruses and the solution-state structure of the nucleocapsid protein from HIV-1. Protein Sci..

[22] Mouhand A., Pasi M., Catala M., Zargarian L., Belfetmi A., Barraud P., Mauffret O., Tisné C. (2020). Overview of the nucleic-acid binding properties of the HIV-1 nucleocapsid protein in its different maturation states. Viruses.

[23] Aldovini A., Young R.A. (1990). Mutations of RNA and protein sequences involved in human immunodeficiency virus type 1 packaging result in production of noninfectious virus. J. Virol..

[24] Gorelick R.J., Nigida S.M., Bess J.W., Arthur L.O., Henderson L.E., Rein A. (1990). Noninfectious human immunodeficiency virus type 1 mutants deficient in genomic RNA. J. Virol..

[25] Abd El-Wahab E.W., Smyth R.P., Mailler E., Bernacchi S., Vivet-Boudou V., Hijnen M., Jossinet F., Mak J., Paillart J.-C., Marquet R. (2014). Specific recognition of the HIV-1 genomic RNA by the Gag precursor. Nat. Commun..

[26] Clever J.L., Miranda D., Parslow T.G. (2002). RNA structure and packaging signals in the 5J leader region of the human immunodeficiency virus type 1 genome. J. Virol..

[27] De Guzman R.N. (1998). Structure of the HIV-1 nucleocapsid protein bound to the SL3 -RNA recognition element. Science.

[28] Lever A., Gottlinger H., Haseltine W., Sodroski J. (1989). Identification of a sequence required for efficient packaging of human immunodeficiency virus type 1 RNA into virions. J. Virol..

[29] Morellet N., Déméné H., Teilleux V., Huynh-Dinh T., de Rocquigny H., Fournié-Zaluski M.-C., Roques B.P. (1998). Structure of the complex between the HIV-1 nucleocapsid protein NCp7 and the single-stranded pentanucleotide d(ACGCC). J. Mol. Biol..

[30] Comas-Garcia M., Datta S.A., Baker L., Varma R., Gudla P.R., Rein A. (2017). Dissection of specific binding of HIV-1 Gag to the “packaging signal” in viral RNA. Elife.

[31] Webb J.A., Jones C.P., Parent L.J., Rouzina I., Musier-Forsyth K. (2013). Distinct binding interactions of HIV-1 Gag to Psi and non-Psi RNAs: Implications for viral genomic RNA packaging. RNA.

[32] Comas-Garcia M., Kroupa T., Datta S.A., Harvin D.P., Hu W.-S., Rein A. (2018). Efficient support of virus-like particle assembly by the HIV-1 packaging signal. Elife.

[33] Dilley K.A., Nikolaitchik O.A., Galli A., Burdick R.C., Levine L., Li K., Rein A., Pathak V.K., Hu W.-S. (2017). Interactions between HIV-1 Gag and viral RNA genome enhance virion assembly. J. Virol..

[34] El Meshri S.E., Dujardin D., Godet J., Richert L., Boudier C., Darlix J.L., Didier P., Mély Y., de Rocquigny H. (2015). Role of the nucleocapsid domain in HIV-1 Gag oligomerization and trafficking to the plasma membrane: A fluorescence lifetime imaging microscopy investigation. J. Mol. Biol..

[35] Chamontin C., Rassam P., Ferrer M., Racine P.-J., Neyret A., Lainé S., Milhiet P.-E., Mougel M. (2015). HIV-1 nucleocapsid and ESCRT-component Tsg101 interplay prevents HIV from turning into a DNA-containing virus. Nucleic Acids Res..

[36] Derdowski A., Ding L., Spearman P. (2004). A novel fluorescence resonance energy transfer assay demonstrates that the human immunodeficiency virus type 1 Pr55Gag I domain mediates Gag-Gag interactions. J. Virol..

[37] Hogue I.B., Hoppe A., Ono A. (2009). Quantitative fluorescence resonance energy transfer microscopy analysis of the human immunodeficiency virus type 1 Gag-Gag interaction: Relative contributions of the CA and NC domains and membrane binding. J. Virol..

[38] Bertrand E., Chartrand P., Schaefer M., Shenoy S.M., Singer R.H., Long R.M. (1998). Localization of ASH1 mRNA particles in living yeast. Mol. Cell.

[39] Ferrer M., Clerté C., Chamontin C., Basyuk E., Lainé S., Hottin J., Bertrand E., Margeat E., Mougel M. (2016). Imaging HIV-1 RNA dimerization in cells by multicolor super-resolution and fluctuation microscopies. Nucleic Acids Res..

[40] Grewe B., Hoffmann B., Ohs I., Blissenbach M., Brandt S., Tippler B., Grunwald T., Uberla K. (2012). Cytoplasmic utilization of human immunodeficiency virus type 1 genomic RNA is not dependent on a nuclear interaction with Gag. J. Virol..

[41] Tuffy K.M., Maldonado R.J.K., Chang J., Rosenfeld P., Cochrane A., Parent L.J. (2020). HIV-1 Gag forms ribonucleoprotein complexes with unspliced viral RNA at transcription sites. Viruses.

[42] Jouvenet N., Bieniasz P.D., Simon S.M. (2008). Imaging the biogenesis of individual HIV-1 virions in live cells. Nature.

[43] Wright E.R., Schooler J.B., Ding H.J., Kieffer C., Fillmore C., Sundquist W.I., Jensen G.J. (2007). Electron cryotomography of immature HIV-1 virions reveals the structure of the CA and SP1 Gag shells. EMBO J..

[44] Olivo-Marin J.-C. (2002). Extraction of spots in biological images using multiscale products. Pattern Recognit..

[45] Gottlinger H.G., Sodroski J.G., Haseltine W.A. (1989). Role of capsid precursor processing and myristoylation in morphogenesis and infectivity of human immunodeficiency virus type 1. Proc. Natl. Acad. Sci. U. S. A..

[46] Pettit S.C., Moody M.D., Wehbie R.S., Kaplan A.H., Nantermet P.V., Klein C.A., Swanstrom R. (1994). The p2 domain of human immunodeficiency virus type 1 Gag regulates sequential proteolytic processing and is required to produce fully infectious virions. J. Virol..

[47] Bazzi A., Zargarian L., Chaminade F., Boudier C., De Rocquigny H., René B., Mély Y., Fossé P., Mauffret O. (2011). Structural insights into the cTAR DNA recognition by the HIV-1 nucleocapsid protein: Role of sugar deoxyriboses in the binding polarity of NC. Nucleic Acids Res..

[48] Amarasinghe G.K., De Guzman R.N., Turner R.B., Summers M.F. (2000). NMR structure of stem-loop SL2 of the HIV-1 psi RNA packaging signal reveals a novel A-U-A base-triple platform. J. Mol. Biol..

[49] Demene H., Dong C.Z., Ottmann M., Rouyez M.C., Jullian N., Morellet N., Mely Y., Darlix J.L., Fournie-Zaluski M.C. (1994). 1H NMR structure and biological studies of the His23-->Cys mutant nucleocapsid protein of HIV-1 indicate that the conformation of the first zinc finger is critical for virus infectivity. Biochemistry.

[50] Beltz H., Clauss C., Piémont E., Ficheux D., Gorelick R.J., Roques B., Gabus C., Darlix J.-L., de Rocquigny H., Mély Y. (2005). Structural determinants of HIV-1 nucleocapsid protein for cTAR DNA binding and destabilization, and correlation with inhibition of self-primed DNA synthesis. J. Mol. Biol..

[51] Darlix J.-L., Lapadat-Tapolsky M., de Rocquigny H., Roques B.P. (1995). First glimpses at structure-function relationships of the nucleocapsid protein of retroviruses. J. Mol. Biol..

[52] Guo J., Wu T., Kane B.F., Johnson D.G., Henderson L.E., Gorelick R.J., Levin J.G. (2002). Subtle alterations of the native zinc finger structures have dramatic effects on the nucleic acid chaperone activity of human immunodeficiency virus type 1 nucleocapsid protein. J. Virol..

[53] Buckman J.S., Bosche W.J., Gorelick R.J. (2003). Human immunodeficiency virus type 1 nucleocapsid Zn(2+) fingers are required for efficient reverse transcription, initial integration processes, and protection of newly synthesized viral DNA. J. Virol..

[54] Didierlaurent L., Houzet L., Morichaud Z., Darlix J.-L., Mougel M. (2008). The conserved N-terminal basic residues and zinc-finger motifs of HIV-1 nucleocapsid restrict the viral cDNA synthesis during virus formation and maturation. Nucleic Acids Res..

[55] Tanchou V., Decimo D., Péchoux C., Lener D., Rogemond V., Berthoux L., Ottmann M., Darlix J.-L. (1998). Role of the N-terminal zinc finger of human immunodeficiency virus type 1 nucleocapsid protein in virus structure and replication. J. Virol..

[56] Gorelick R.J., Gagliardi T.D., Bosche W.J., Wiltrout T.A., Coren L.V., Chabot D.J., Lifson J.D., Henderson L.E., Arthur L.O. (1999). Strict conservation of the retroviral nucleocapsid protein zinc finger is strongly influenced by its role in viral infection processes: Characterization of HIV-1 particles containing mutant nucleocapsid zinc-coordinating sequences. Virology.

[57] Tanwar H.S., Khoo K.K., Garvey M., Waddington L., Leis A., Hijnen M., Velkov T., Dumsday G.J., McKinstry W.J., Mak J. (2017). The thermodynamics of Pr55Gag-RNA interaction regulate the assembly of HIV. PLoS Pathog..

[58] Wang W., Naiyer N., Mitra M., Li J., Williams M.C., Rouzina I., Gorelick R.J., Wu Z., Musier-Forsyth K. (2014). Distinct nucleic acid interaction properties of HIV-1 nucleocapsid protein precursor NCp15 explain reduced viral infectivity. Nucleic Acids Res..

[59] Dubois N., Khoo K.K., Ghossein S., Seissler T., Wolff P., McKinstry W.J., Mak J., Paillart J.-C., Marquet R., Bernacchi S. (2018). The C-terminal p6 domain of the HIV-1 Pr55^Gag^ precursor is required for specific binding to the genomic RNA. RNA Biol..

[60] Sarni S., Biswas B., Liu S., Olson E.D., Kitzrow J.P., Rein A., Wysocki V.H., Musier-Forsyth K. (2020). HIV-1 Gag protein with or without p6 specifically dimerizes on the viral RNA packaging signal. J. Biol. Chem..

[61] Huang M., Orenstein J.M., Martin M.A., Freed E.O. (1995). p6Gag is required for particle production from full-length human immunodeficiency virus type 1 molecular clones expressing protease. J. Virol..

[62] Freed E.O. (2003). The HIV–TSG101 interface: Recent advances in a budding field. Trends Microbiol..

[63] Rose K.M., Hirsch V.M., Bouamr F. (2020). Budding of a retrovirus: Some assemblies required. Viruses.

[64] Rein A. (2019). RNA packaging in HIV. Trends Microbiol..

[65] Berkhout B., van Wamel J.L. (1996). Role of the DIS hairpin in replication of human immunodeficiency virus type 1. J. Virol..

[66] Hussein I.T.M., Ni N., Galli A., Chen J., Moore M.D., Hu W.-S. (2010). Delineation of the preferences and requirements of the human immunodeficiency virus type 1 dimerization initiation signal by using an *in vivo* cell-based selection approach. J. Virol..

[67] Laughrea M., Jetté L., Mak J., Kleiman L., Liang C., Wainberg M.A. (1997). Mutations in the kissing-loop hairpin of human immunodeficiency virus type 1 reduce viral infectivity as well as genomic RNA packaging and dimerization. J. Virol..

[68] Paillart J.C., Skripkin E., Ehresmann B., Ehresmann C., Marquet R. (1996). A loop-loop “kissing” complex is the essential part of the dimer linkage of genomic HIV-1 RNA. Proc. Natl. Acad. Sci. U. S. A..

[69] Skripkin E., Paillart J.C., Marquet R., Ehresmann B., Ehresmann C. (1994). Identification of the primary site of the human immunodeficiency virus type 1 RNA dimerization *in vitro*. Proc. Natl. Acad. Sci. U. S. A..

[70] Didierlaurent L., Racine P.J., Houzet L., Chamontin C., Berkhout B., Mougel M. (2011). Role of HIV-1 RNA and protein determinants for the selective packaging of spliced and unspliced viral RNA and host U6 and 7SL RNA in virus particles. Nucleic Acids Res..

[71] Houzet L., Paillart J.C., Smagulova F., Maurel S., Morichaud Z., Marquet R., Mougel M. (2007). HIV controls the selective packaging of genomic, spliced viral and cellular RNAs into virions through different mechanisms. Nucleic Acids Res..

[72] Luban J., Goff S.P. (1994). Mutational analysis of cis-acting packaging signals in human immunodeficiency virus type 1 RNA. J. Virol..

[73] Poole E., Strappe P., Mok H.-P., Hicks R., Lever A.M.L. (2005). HIV-1 Gag-RNA interaction occurs at a perinuclear/centrosomal site; analysis by confocal microscopy and FRET: HIV-1 Gag-RNA interaction occurs in a perinuclear region. Traffic.

[74] Chen J., Rahman S.A., Nikolaitchik O.A., Grunwald D., Sardo L., Burdick R.C., Plisov S., Liang E., Tai S., Pathak V.K., Hu W.-S. (2016). HIV-1 RNA genome dimerizes on the plasma membrane in the presence of Gag protein. Proc. Natl. Acad. Sci. U. S. A..

[75] Sardo L., Hatch S.C., Chen J., Nikolaitchik O., Burdick R.C., Chen D., Westlake C.J., Lockett S., Pathak V.K., Hu W.-S. (2015). Dynamics of HIV-1 RNA near the plasma membrane during virus assembly. J. Virol..

[76] Ivanchenko S., Godinez W.J., Lampe M., Kräusslich H.-G., Eils R., Rohr K., Bräuchle C., Müller B., Lamb D.C. (2009). Dynamics of HIV-1 assembly and release. PLoS Pathog..

[77] Milev M.P., Brown C.M., Mouland A.J. (2010). Live cell visualization of the interactions between HIV-1 Gag and the cellular RNA-binding protein Staufen1. Retrovirology.

[78] Maldonado R.J.K., Rice B., Chen E.C., Tuffy K.M., Chiari E.F., Fahrbach K.M., Hope T.J., Parent L.J. (2020). Visualizing association of the retroviral Gag protein with unspliced viral RNA in the nucleus. mBio.

[79] Ricaña C.L., Johnson M.C. (2021). An infectious rous sarcoma virus Gag mutant that is defective in nuclear cycling. J. Virol..

[80] Mougel M., Akkawi C., Chamontin C., Feuillard J., Pessel-Vivares L., Socol M., Laine S. (2020). NXF1 and CRM1 nuclear export pathways orchestrate nuclear export, translation and packaging of murine leukaemia retrovirus unspliced RNA. RNA Biol..

[81] Rulli S.J., Hibbert C.S., Mirro J., Pederson T., Biswal S., Rein A. (2007). Selective and nonselective packaging of cellular RNAs in retrovirus particles. J. Virol..

[82] Chen J., Nikolaitchik O., Singh J., Wright A., Bencsics C.E., Coffin J.M., Ni N., Lockett S., Pathak V.K., Hu W.-S. (2009). High efficiency of HIV-1 genomic RNA packaging and heterozygote formation revealed by single virion analysis. Proc. Natl. Acad. Sci. U. S. A..

[83] Gorelick R.J., Chabot D.J., Rein A., Henderson L.E., Arthur L.O. (1993). The two zinc fingers in the human immunodeficiency virus type 1 nucleocapsid protein are not functionally equivalent. J. Virol..

[84] Schwartz M.D., Fiore D., Panganiban A.T. (1997). Distinct functions and requirements for the Cys-His boxes of the human immunodeficiency virus type 1 nucleocapsid protein during RNA encapsidation and replication. J. Virol..

[85] Retureau R., Oguey C., Mauffret O., Hartmann B. (2019). Structural explorations of NCp7–nucleic acid complexes give keys to decipher the binding process. J. Mol. Biol..

[86] Zargarian L., Tisné C., Barraud P., Xu X., Morellet N., René B., Mély Y., Fossé P., Mauffret O. (2014). Dynamics of linker residues modulate the nucleic acid binding properties of the HIV-1 nucleocapsid protein zinc fingers. PLoS One.

[87] Lever A.M.L. (2007). HIV-1 RNA packaging. Adv. Pharmacol..

[88] Berkhout B., Van Wamel J.L.B. (2000). The leader of the HIV-1 RNA genome forms a compactly folded tertiary structure. RNA.

[89] Haddrick M., Lear A.L., Cann A.J., Heaphy S. (1996). Evidence that a kissing loop structure facilitates genomic RNA dimerisation in HIV-1. J. Mol. Biol..

[90] Hill M.K., Shehu-Xhilaga M., Campbell S.M., Poumbourios P., Crowe S.M., Mak J. (2003). The dimer initiation sequence stem-loop of human immunodeficiency virus type 1 is dispensable for viral replication in peripheral blood mononuclear cells. J. Virol..

[91] Shen N., Jetté L., Liang C., Wainberg M.A., Laughrea M. (2000). Impact of human immunodeficiency virus type 1 RNA dimerization on viral infectivity and of stem-loop B on RNA dimerization and reverse transcription and dissociation of dimerization from packaging. J. Virol..

[92] Ding P., Kharytonchyk S., Waller A., Mbaekwe U., Basappa S., Kuo N., Frank H.M., Quasney C., Kidane A., Swanson C., Van V., Sarkar M., Cannistraci E., Chaudhary R., Flores H. (2020). Identification of the initial nucleocapsid recognition element in the HIV-1 RNA packaging signal. Proc. Natl. Acad. Sci. U. S. A..

[93] Russell R.S., Hu J., Beriault V., Mouland A.J., Kleiman L., Wainberg M.A., Liang C. (2003). Sequences downstream of the 5J splice donor site are required for both packaging and dimerization of human immunodeficiency virus type 1 RNA. J. Virol..

[94] Kaye J.F., Richardson J.H., Lever A.M. (1995). Cis-acting sequences involved in human immunodeficiency virus type 1 RNA packaging. J. Virol..

[95] Kutluay S.B., Zang T., Blanco-Melo D., Powell C., Jannain D., Errando M., Bieniasz P.D. (2014). Global changes in the RNA binding specificity of HIV-1 Gag regulate virion genesis. Cell.

[96] Monette A., Niu M., Chen L., Rao S., Gorelick R.J., Mouland A.J. (2020). Pan-retroviral nucleocapsid-mediated phase separation regulates genomic RNA positioning and trafficking. Cell Rep..

[97] Querido E., Chartrand P. (2008). Using fluorescent proteins to study mRNA trafficking in living cells. Methods Cell Biol..

[98] Fusco D., Accornero N., Lavoie B., Shenoy S.M., Blanchard J.-M., Singer R.H., Bertrand E. (2003). Single mRNA molecules demonstrate probabilistic movement in living mammalian cells. Curr. Biol..

[99] Querido E., Gallardo F., Beaudoin M., Menard C., Chartrand P. (2011). Stochastic and reversible aggregation of mRNA with expanded CUG-triplet repeats. J. Cell Sci..

