# Supplementary material for: Quantitative analysis of the formation of nucleoprotein complexes between HIV-1 Gag protein and genomic RNA using transmission electron microscopy
Source: J Biol Chem. 2021 Dec 17;298(1):101500. doi: 10.1016/j.jbc.2021.101500 (PMC8760521; doi:10.1016/j.jbc.2021.101500)
Supplement: Supplemental Figures S1–S5 [file mmc1.docx]

**Supporting information**

**Quantitative analysis of the formation of nucleoprotein complexes between HIV-1 Gag protein and genomic RNA using transmission electron microscopy**

^1^Stéphanie Durand, ^1^Florian Seigneuret, ^2^Julien Burlaud-Gaillard, ^3^Roxane Lemoine, ^1^Marc-Florent Tassi, ^1^Alain Moreau, ^5^Marylène Mougel, ^1,2^Philippe Roingeard, ^4^Clovis Tauber, and ^1^Hugues de Rocquigny*

Gag was detected by an anti-p24 antibody revealed by an anti-mouse-ALEXA 594 for confocal microscopy (Fig. S1A, image a) or by anti-mouse decorated by 10 nm gold beads for TEM (Fig. S1A, image b, insets 1 and 2). Sometimes unlabeled virions were detected on TEM images (Fig. S1A, image b, inset 2, blue arrow). The pMCP-eGFP-NLS plasmid encodes for the MS2-Coat-Protein (MCP) fused to eGFP and including a nuclear localization signal (NLS) (1, 2). This chimeric protein was mainly localized in the nucleus when viewed by eGFP fluorescent emission (Fig. S1A, image c) and by TEM using the 6 nm beads labelling the anti-eGFP antibody (Fig. S1A, image d, insets 3 and 4). In the presence of the gRNA-MS2-Δenv, the MCP-eGFP-NLS protein was mainly in the cytoplasm (Fig. S1A, images e and f, insets 5 and 6) (1).

To ensure that gold beads corresponded to the desired target, passivation and washing of cryo-sections were performed (see Experimental procedures). First, naive Hela cells were incubated with anti-p24 and anti-eGFP antibodies and then with gold conjugated goat-anti-mouse (10 nm) and gold conjugated goat-anti-rabbit (6 nm) antibodies (Fig. S1B, image a). The scale is 1 µm by 1 µm to observe a larger surface. On these images, rare 10 nm gold beads were distinguished (Fig. S1B, image a, full white arrows) and no 6 nm gold beads were detected. Another control was performed with Hela cells transfected with the pNL4.3-MS2-Δenv plasmid and stained only with the two gold conjugated secondary antibodies (Fig. S1B, image b). Here again, only a few 10 nm beads were observed mostly located on the plate (Fig. S1B, image a, full white arrows). Other electron dense structures were sometime found on electron micrographs but are not related to the gold labelling (Fig. S1B, image a, empty white arrows). Thus, the signals observed in our TEM images undoubtedly correspond to Gag and viral gRNA.


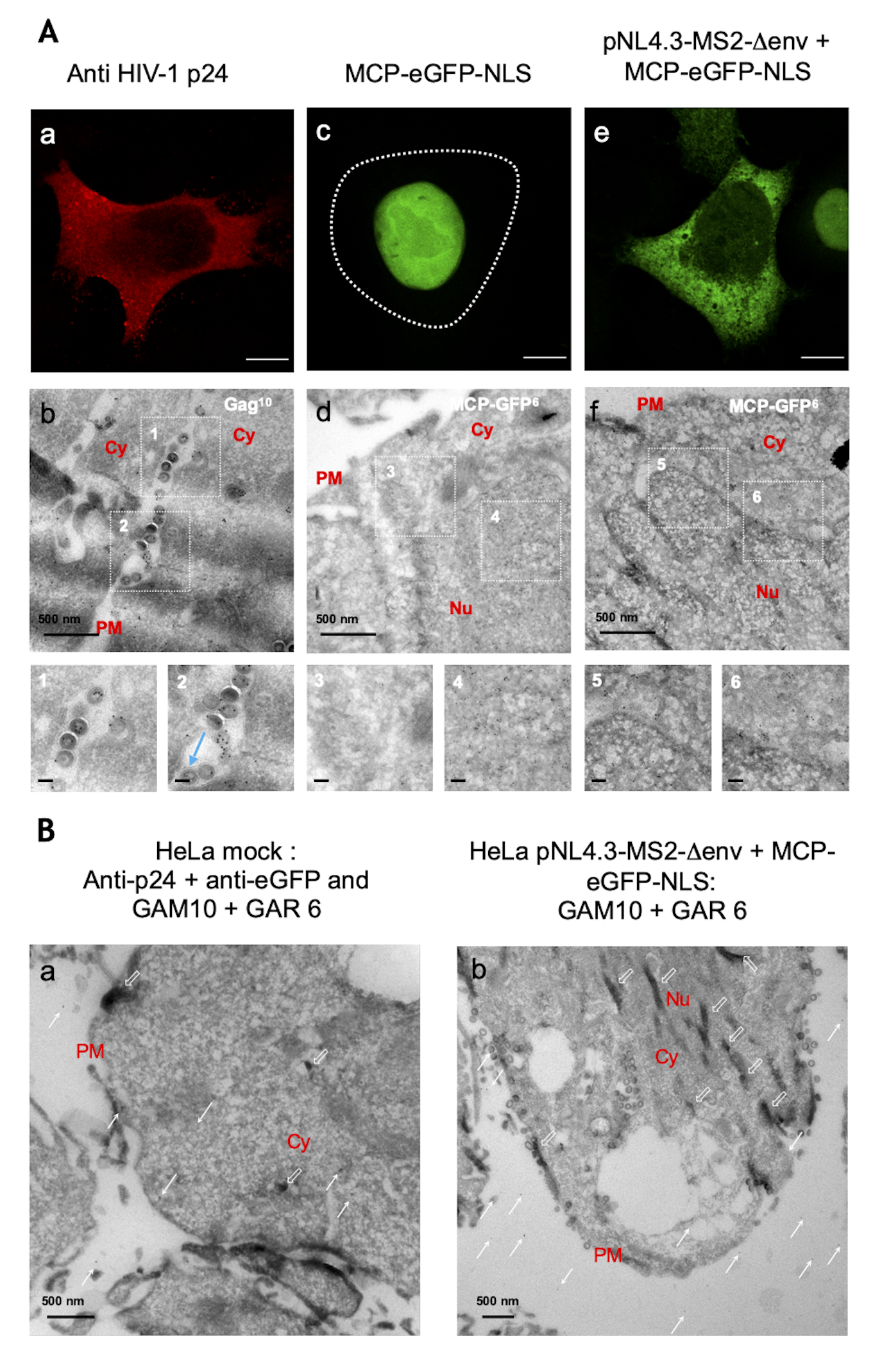


**Figure S1. A:** Expression of HIV-1 Gag (a, b), MCP-eGFP-NLS (c, d) and pNL4.3-MS2-Δenv together with MCP-eGFP-NLS (e, f) in HeLa cells 24h PT and observed by confocal microscopy (a, c, e) and TEM (b, d, f). For confocal microscopy, Gag was detected by an anti-p24 antibody and gRNA-MS2-Δenv by the eGFP fluorescence. For TEM, cells were GFP-based sorted, fixed by 4% PFA and 0.1% glutaraldehyde, embedded in gelatin and cryo-protected in 2.3M sucrose. Ultrathin sections were cutted and incubated first either with a mouse anti-p24 or a rabbit anti-eGFP antibody and then detected with anti-mouse or anti-rabbit antibodies conjugated with 10 nm or 6 nm gold beads respectively. The blue arrow on inset 2 represent a non-labelled VLP, sometimes observed on our electron micrographs. Scale bar in white corresponds to 10 µm. B: Controls of the antibodies used for TEM. HeLa cells were treated for TEM as for A. a: Naïve HeLa cells were stained with anti-p24 and anti-eGFP antibodies followed by goat-anti-mouse 10 nm (GAM10) and goat-anti-rabbit 6 nm (GAR6) antibodies respectively. b: HeLa cells transfected with a mixture of pNL4.3-MS2-Δenv and pMCP-eGFP-NLS were only treated with gold-conjugated secondary antibodies. Nu: Nucleus. Cy: Cytoplasm. PM: Plasma Membrane. Full white arrows correspond to 10 nm beads while empty white arrows represent electron dense structure. Scale bar in black for zoom magnification corresponds to 100nm.


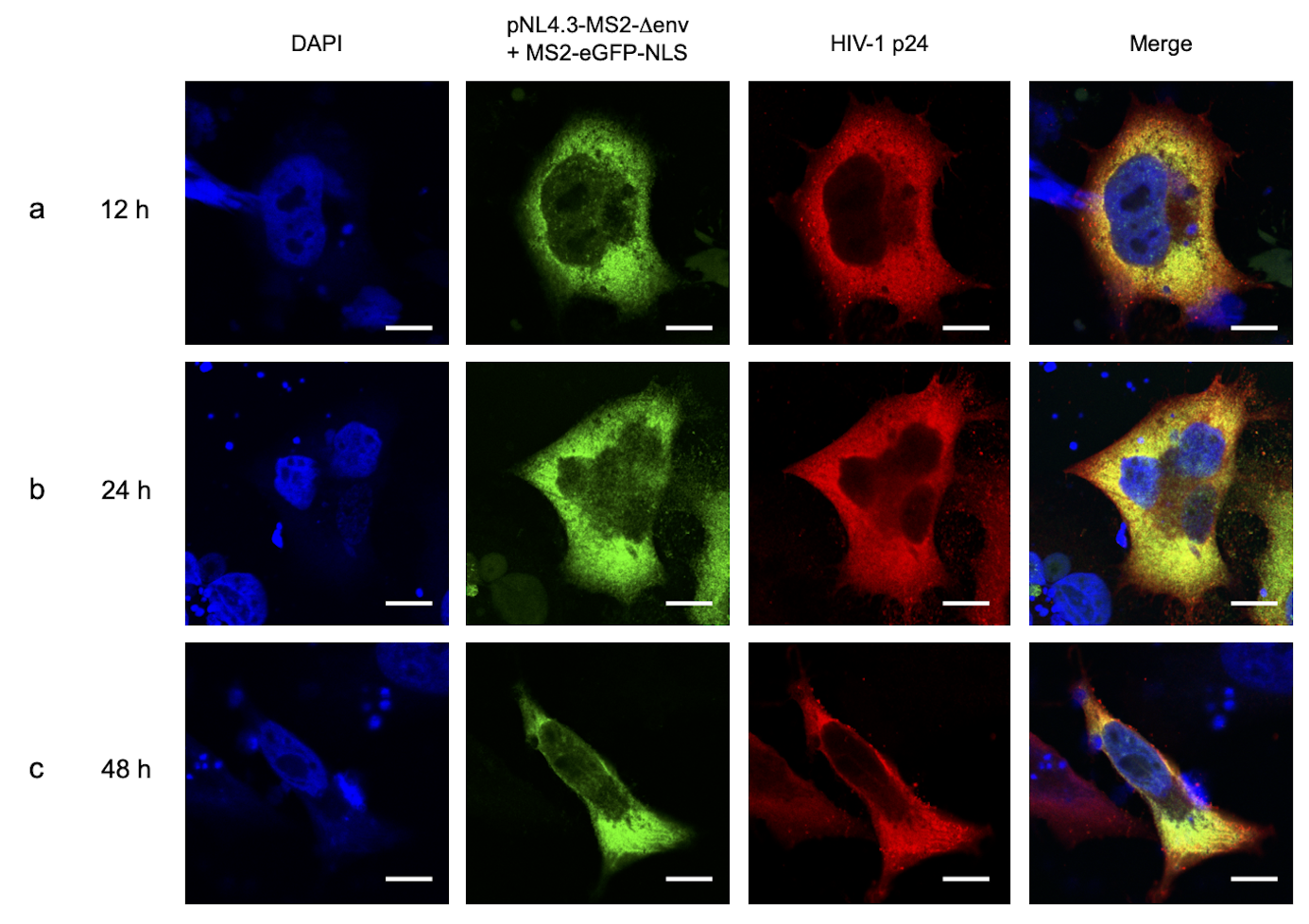


**Figure S2.** Observation of the co-localization between Gag and gRNA-MS2-Δenv as function of the time by confocal microscopy. HeLa cells were transiently co-transfected with a mixture of DNA plasmids expressing gRNA-MS2-Δenv and MCP-eGFP-NLS protein (Ratio 0.6/0.4). After 12 h, 24 h or 48 h of expression, Gag was detected by an anti-p24 antibody and gRNA-MS2-Δenv by the eGFP fluorescence as described in Fig. S1A. Each panel shows the major observed phenotype. Nucleus is detected by DAPI staining in blue, MCP-eGFP-NLS protein is in green and Gag protein is in red. Scale bar in white corresponds to 10 µm.


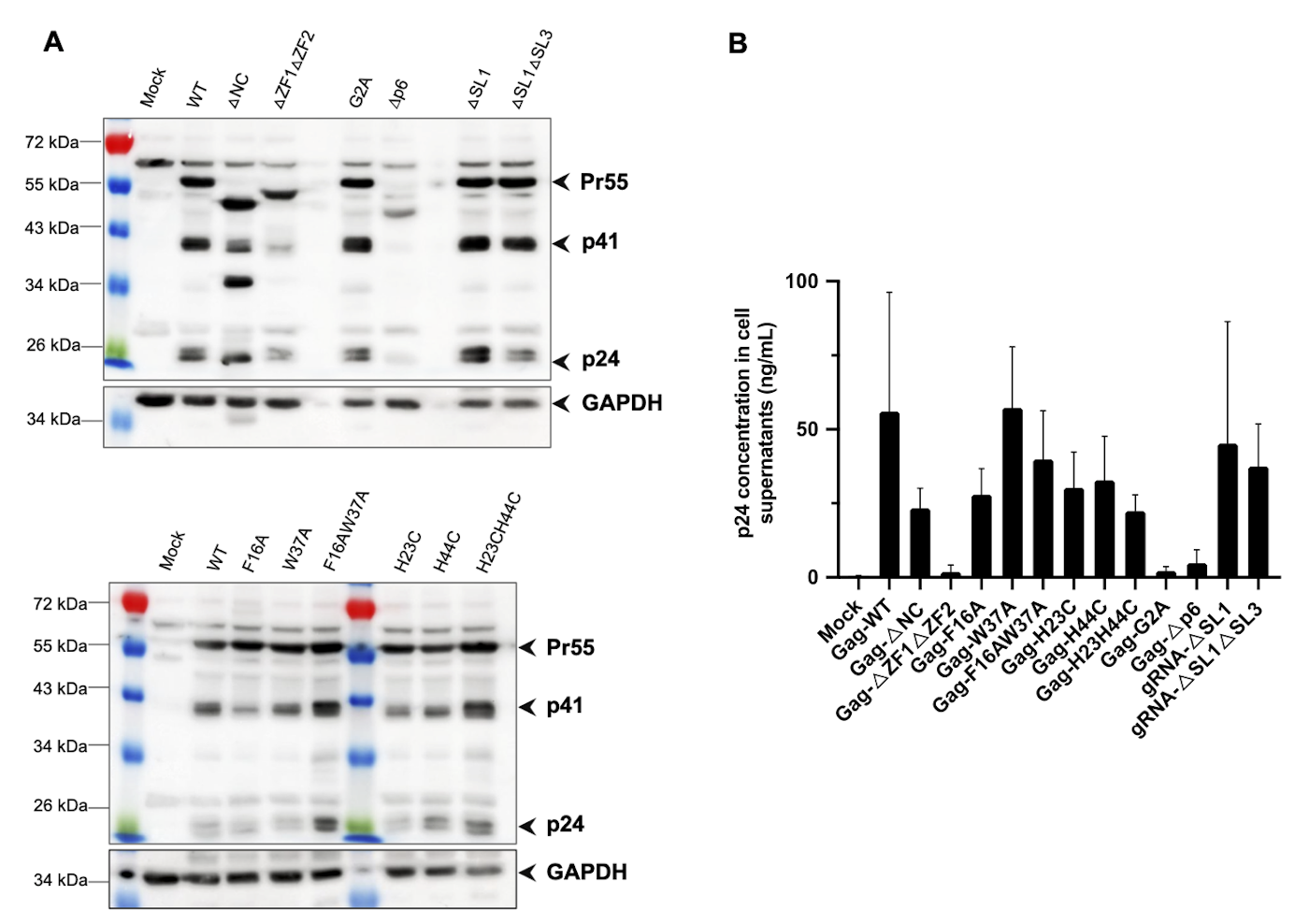


**Figure S3.** Intracellular and extracellular HIV-1 Gag and Gag derivates expression level. HeLa cells were transfected with plasmids pNL4.3-MS2-Δenv expressing either the Gag-WT or derivates. One day after transfection, cells were analyzed by Western blotting for Gag expression and secretion. Different maturation forms of Gag (p55, p41, p24) are referred. A: Intracellular Gag protein analysis by Western Blot. Cell lysates were separated by SDS-PAGE, the proteins were transferred onto PVDF membranes and probed with anti-HIV-1 p24 (high panel) or anti-GAPDH (bottom panel) antibodies. The GAPDH housekeeping protein was used to normalize protein loading between lanes. **B:** Extracellular Gag protein analysis. Cell supernatants were purified on a sucrose cushion and p24 concentration was analyzed by quantitative ELISA.


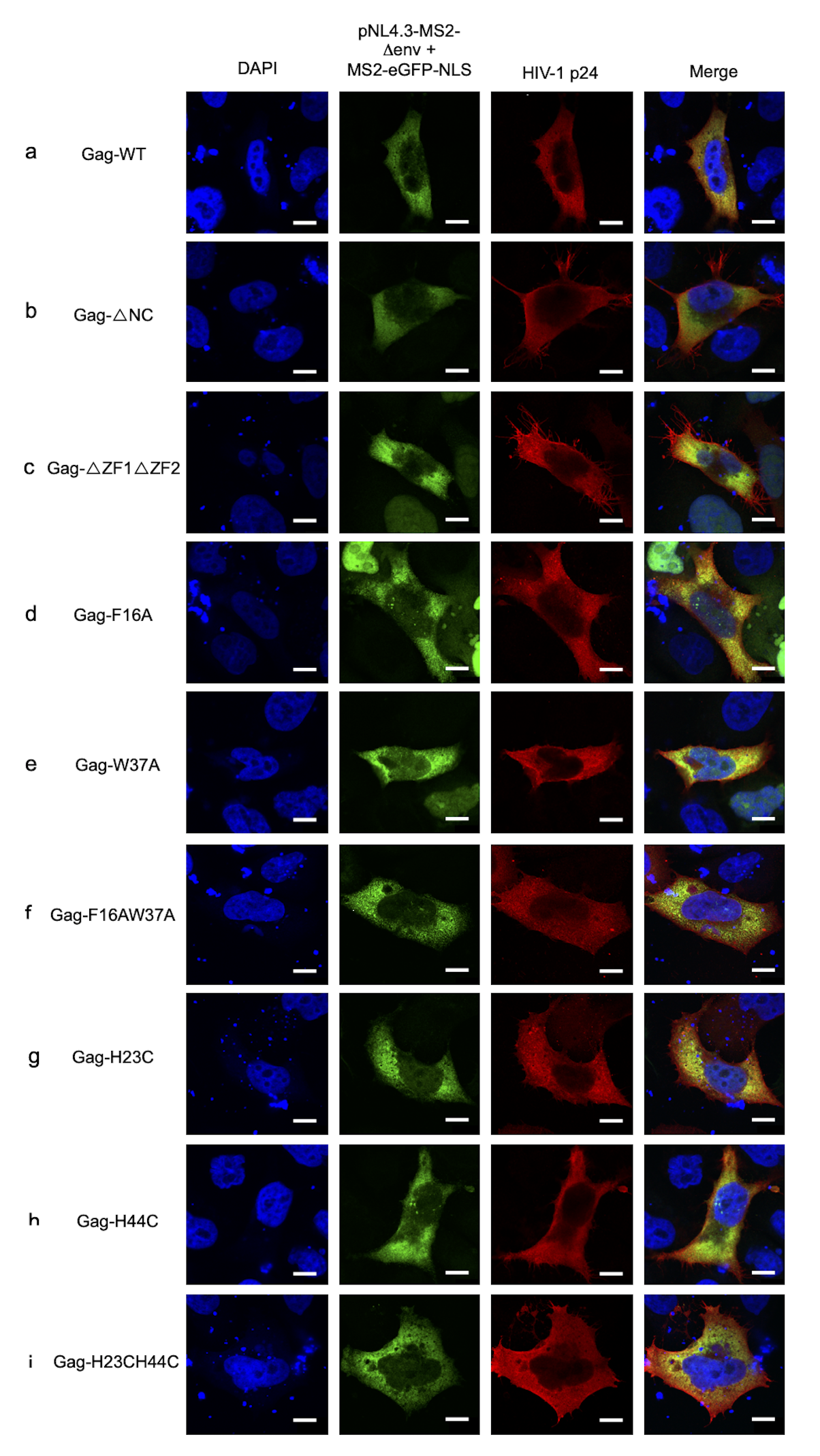


**Figure S4.** Impact of NC deletions or ZF punctual mutations on Gag and gRNA-MS2-Δenv co-localization, monitored by confocal microscopy. HeLa cells were co-transfected with modified pNL4.3-MS2-Δenv encoding for Gag (a), Gag-ΔNC (b), Gag-ΔZF1ΔZF2 (c), Gag-F16A (d), Gag-W37A (e), Gag-F16AW37A (f), Gag-H23C (g), Gag-H44C (h) or Gag-H23CH44C (i) and a plasmid expressing the MCP-eGFP-NLS protein. Cells were observed 24h PT and Gag was detected by an anti-p24 antibody and gRNA-MS2-Δenv by the eGFP fluorescence. Each panel shows the major observed phenotype. Nucleus is detected by DAPI staining in blue, MCP-eGFP-NLS protein is in green and Gag protein is in red. Scale bar in white corresponds to 10 µm.


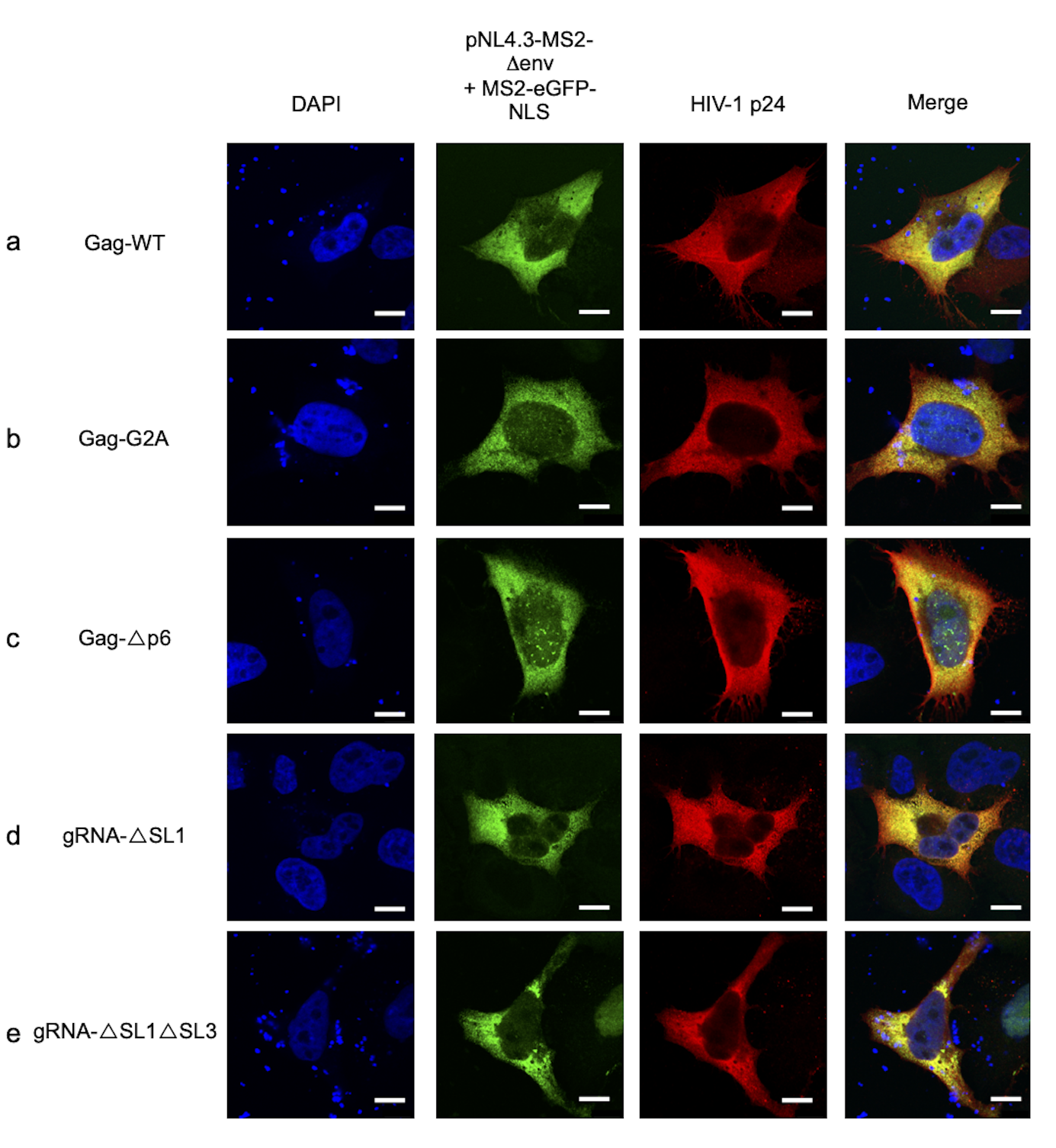


**Figure S5**. Impact of Gag or gRNA mutations on Gag-gRNA co-localization, monitored by confocal microscopy. HeLa cells were co-transfected with modified pNL4.3-MS2-Δenv encoding for Gag (a), Gag-G2A (b), Gag-Δp6 (c), gRNA-ΔSL1 (d) or gRNA-ΔSL1ΔSL3 (e) and a plasmid expressing the MCP-eGFP-NLS. Cells were observed 24h PT and Gag was detected by an anti-p24 antibody and gRNA-MS2-Δenv by the eGFP fluorescence. Each panel shows the major observed phenotype. Nucleus is detected by DAPI staining in blue, MCP-eGFP-NLS protein is in green and Gag protein is in red. Scale bar in white corresponds to 10 µm.

**References**

1. Fusco, D., Accornero, N., Lavoie, B., Shenoy, S. M., Blanchard, J.-M., Singer, R. H., and Bertrand, E. (2003) Single mRNA Molecules Demonstrate Probabilistic Movement in Living Mammalian Cells. *Curr. Biol.* 13, 161–167

2. Querido, E., Gallardo, F., Beaudoin, M., Menard, C., and Chartrand, P. (2011) Stochastic and reversible aggregation of mRNA with expanded CUG-triplet repeats. *J. Cell Sci.* 124, 1703–1714
